# Robustness Analysis of Stochastic Biochemical Systems

**DOI:** 10.1371/journal.pone.0094553

**Published:** 2014-04-21

**Authors:** Milan Česka, David Šafránek, Sven Dražan, Luboš Brim

**Affiliations:** Systems Biology Laboratory at Faculty of Informatics, Masaryk University, Brno, Czech Republic; University of Bonn, Bonn-Aachen International Center for IT, Germany

## Abstract

We propose a new framework for rigorous robustness analysis of stochastic biochemical systems that is based on probabilistic model checking techniques. We adapt the general definition of robustness introduced by Kitano to the class of stochastic systems modelled as continuous time Markov Chains in order to extensively analyse and compare robustness of biological models with uncertain parameters. The framework utilises novel computational methods that enable to effectively evaluate the robustness of models with respect to quantitative temporal properties and parameters such as reaction rate constants and initial conditions. We have applied the framework to gene regulation as an example of a central biological mechanism where intrinsic and extrinsic stochasticity plays crucial role due to low numbers of DNA and RNA molecules. Using our methods we have obtained a comprehensive and precise analysis of stochastic dynamics under parameter uncertainty. Furthermore, we apply our framework to compare several variants of two-component signalling networks from the perspective of robustness with respect to intrinsic noise caused by low populations of signalling components. We have successfully extended previous studies performed on deterministic models (ODE) and showed that stochasticity may significantly affect obtained predictions. Our case studies demonstrate that the framework can provide deeper insight into the role of key parameters in maintaining the system functionality and thus it significantly contributes to formal methods in computational systems biology.

## Introduction

Robustness is one of the fundamental features of biological systems. According to Kitano [1] *“robustness is a property that allows a system to maintain its functions against internal and external perturbations”*. To formally analyse robustness, we must thus precisely define a model of a biological system, its perturbations and the notions of a system’s function. In this paper, we propose a novel framework for robustness analysis of stochastic biochemical systems. To this end, inspected systems are described by means of stochastic biochemical kinetic models, system’s functionality is defined by its logical properties, and system’s perturbation is modelled as a change in stochastic kinetic parameters or initial conditions of the model.

Processes occurring inside living cells exhibit dynamics that can be observed and classified as carrying out a certain function – maintaining stable concentrations, responding to an environment change, growth, etc. Kinetic models with parameters are used to formally capture cell dynamics. Limited knowledge of numerical parameters poses a challenge since precise values of all parameters (kinetic constants, initial concentrations, environmental conditions, etc.) may be unknown, may be known but imprecisely, or may in principle form a bounded uncertainty interval (e.g., non-homogeneous cell populations, different structural conformations of a molecule leading to multiple kinetic rates, etc.). Hence, the behaviour of a kinetic model for a given single parametric instantiation and its derived functionality may not provide an adequate result. Therefore it is necessary to take into account possible uncertainties, variance and inhomogeneities.

The concept of robustness addresses this aspect of functional evaluation by considering a weighted average of every behaviour across a space of perturbations, each altering the model parameters (hence its behaviour) and in a particular way, having a certain probability of occurrence. A general definition of robustness, as introduced by Kitano [2], gives us *robustness degree* that quantitatively characterises to what extent is the evaluated system functionality preserved under considered perturbations:
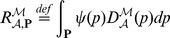
where 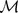 is the system, 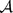 is the function under scrutiny, 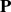 is the space of all perturbations, 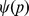 is the probability of the perturbation 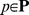 and 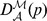 is an *evaluation function* stating how much the function 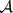 is preserved under a perturbation *p* in the system 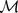.

For the macroscopic view as provided by the deterministic modelling framework based on ordinary differential equations (ODEs), the concept of robustness has been widely studied. There exist several well-established analytic techniques based on static analysis as well as dynamic numerical methods for effective robustness analysis of ODE models. In circumstances of low molecular/cellular numbers such as in signalling [3], immunity reactions or gene regulation [4], intrinsic and extrinsic noise plays an important role and thus these processes are more faithfully modelled stochastically.

In our work, we consider stochastic biochemical kinetic models with the semantics given by *Continuous Time Markov Chains* (CTMCs). The evolution of a probability density vector (further denoted as a *distribution*) describing populations of particular species is given by the chemical master equation (CME) [5]. A function of a system in the biological sense is any intuitively understandable behaviour, e.g., stability of *ERK* signal effector population in high concentration is observed during first 10 minutes. In order to define the robustness of a system formally we need to make precise the intuitive and informal concept of functionality. Our framework builds on the formal methods where the function of a system is expressed indirectly by its logical properties. This leads to a more abstract approach emphasising the most relevant aspects of a system function and suppressing less important technicalities. We use stochastic temporal logics, namely the bounded time fragment of *Continuous Stochastic Logic (CSL)* [6] further extended with *rewards* [7]. The aforementioned example of the behaviour can be formalised using the CSL formula 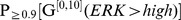 that expresses the property “The probability that the population of 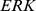 remains in high concentration during first 10 minutes is greater than 90%”. To broaden the scope of possibly captured functionalities we extend CSL with a class of *post-processing functions* defined over probability density vectors. We show that the bounded fragment of CSL with rewards and post-processing functions can adequately capture many biologically relevant scenarios observed in a finite time horizon.

The main methodological contribution of this paper is the adaptation of the concept of robustness to stochastic systems. The main challenge of such adaptation lies in the interpretation of the evaluation function 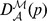. We discuss several definitions of the evaluation function that give us different options how to quantify the ability of the system to preserve the inspected functionality under parameter perturbation. We show how the robustness of stochastic systems can be analysed using the proposed framework that is based on our recently published numerical approximation of the evaluation function [8]. In contrast to existing methods employing parameter sampling and statistical techniques our approximation provides accuracy guarantees.

We apply the framework to two relevant biological problems from the area of cellular processes where stochasticity is inherent and where it plays a crucial role, especially due to low numbers of molecules involved. First, we analyse a model predicting dynamics of a gene regulatory circuit controlling the 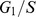 phase transition in the cell cycle of mammalian cells. Stochasticity of the gene regulation becomes critical especially when dealing with genetic switches that make irreversible decisions in tissue development or cell cycle control processes. Without studying the distribution of cell population with respect to the probability of decisions they make, we cannot analyse how robust the decisions are and how certain parameters affect them. Second, we study two models representing different topologies of a general two-component signalling mechanism present in procaryotic cells. Cell signalling is another phenomenon amenable to stochasticity. In state-of-the-art medicine it is necessary to study signalling pathways from the perspective of robust signal response. The notion of robustness is in this case understood in terms of the amount of noise produced in signal response. The lower the level of noise, the more robust is the signal response. We show that our framework provides deeper understanding of how the validity of an inspected hypothesis depends on reaction rate parameters and initial conditions.

The first case study exploits the usability of the method to analyse bistability (and its robustness) in the stochastic framework and thus provides a stochastic analysis analogy to the study presented in [9] under the deterministic (ODE) setting. Robustness is employed to characterise parameterisations of the model with respect to the tendency of the molecule population to choose one of the possible steady states, irreversibly deciding whether the cell will or will not commit to *S*-phase. The results show that intrinsic and extrinsic noise, caused by randomness in protein-DNA binding/unbinding events and other processes controlling the chemical affinity of involved molecules, can significantly affect the cell decision. In our model, the intrinsic noise of chemical reactions is inherently captured by stochastic mass action kinetics whereas the extrinsic noise is considered by means of parameter uncertainty.

The second case study focuses on analysing the effect of intrinsic noise on the signalling pathway functionality. In particular, two topologically different variants of a two-component signalling pathway are exploited for different levels of input signal and different levels of intrinsic noise appearing in transcription of the two signalling components. The considered topologies have been compared in the previous study presented by Steuer et al. [10], where robustness has been analysed in the setting of deterministic (ODE) models. Here the signalling mechanism is remodelled in the stochastic setting and robustness is employed to quantify under which circumstances the individual topologies are less amenable to intrinsic noise of the underlying protein transcription mechanism. The results show that the stochastic approach can uncover facts unpredictable in the deterministic setting.

Formal analysis of complex stochastic biological systems employing both the numerical and the statistical methods generally suffers from extremely high computational demands. These computational demands are even more critical if we need to analyse systems with uncertain parameters, which is also the case of our framework. However, our framework has been designed to be adapted to high-performance computing platforms (e.g., multi-core workstations and massively parallel general-purpose graphic processing units) and also to be successfully combined with existing acceleration methods described in, e.g., [11]–[13]. Although the acceleration is a subject of our future research (inspired by our previous results [14]), we already employ the fact that the approximation method can be efficiently parallelised. In the second case study, where the analysis of the inspected perturbation space requires an extensive numerical computation, we utilise a high-performance multi-core workstation to achieve the acceleration.

The main contributions of this paper can be summarised in the following way:

1. Adaptation of the general concept of robustness of Kitano [2] to the class of stochastic systems modelled by CTMCs.
2. Introduction of a novel framework based on formal methods to evaluate robustness of the stochastic system with respect to the functionality given by a stochastic temporal property and to perturbations of reaction rate parameters and initial conditions.
3. Experimental results providing detailed analysis of stochasticity and parameter uncertainty in mammalian cell cycle gene regulation and robustness analysis of different topologies of two-component signalling systems incorporating stochastic noise.

### Related Work

The discussion of related work can be roughly divided into two parts. First, we summarise the existing methods for parameter exploration and robustness analysis of stochastic models. Second, we briefly mention the methods and tools allowing for robustness analysis of ODE models.

The main goal of our framework is to analyse how the validity of an *a priori* given hypothesis expressed as a temporal property depends on uncertain parameters of the inspected stochastic system. For this purpose we adapt the general definition of robustness [2] to the class of stochastic systems. While the concept of robustness is well established for deterministic systems [15], [16], it has not been adequately addressed for stochastic systems. The key difference is the fact that evolution of a stochastic system is given by a set of paths in contrast to a single trajectory as in the case of a deterministic system. Hence a stochastic system at any given time is described by a probability distribution over states of the corresponding CTMC in contrast to the single state representation of a deterministic system. Therefore, the definition of robustness for stochastic systems requires a more sophisticated interpretation of the evaluation function that determines how the quantitative temporal property is preserved under a perturbation of the system parameters.

Owing to the reasons mentioned in the previous paragraph, parameter estimation methods and the concept of robustness are not yet as established for stochastic models as in the case of ODE models. We have recently published a method [8] where the CSL model checking techniques are extended in order to systematically explore the parameters of stochastic biochemical kinetic models. In [17] a CTMC is explored with respect to a property formalised as a deterministic timed automaton (DTA). It extends [18] to parameter estimation with respect to the acceptance of the DTA. Most approaches to parameter estimation [18]–[20] rely on approximating the maximum likelihood. Their advantage is the possibility to analyse infinite state spaces [18] (employing dynamic state space truncation with numerically computed likelihood) or even models with no prior knowledge of parameter ranges [20] (using Monte-Carlo optimisation for computing the likelihood). In [21] the moment closure approach is considered to capture the distribution of highly populated species in combination with discrete stochastic description for low populated species. The method is able to cope with multi-modal distributions appearing in multi-stable systems. The method introduced in [22] exploits fluid (limit) approximation techniques, enabling an alternative approach to CSL model checking of stochastic models. Despite the computational efficiency, a shared disadvantage of all the mentioned methods is that they rely on approximations applicable only to models that include highly populated species. This is not the case of, e.g., gene regulation dynamics.

Approaches based on Markov Chain Monte-Carlo sampling and Bayesian inference [23]–[25] can be extended to sample-based approximation of the evaluation function, but at the price of undesired inaccuracy and high computational demands [26], [27]. Compared to these methods, our method provides the upper and lower bounds of the result, which makes it more reliable and precise but at the price of even higher computational demands. The most relevant contribution to this domain has been recently introduced by Bartocci et al. [28]. To the best of our knowledge, this is the only related work addressing robustness of stochastic biochemical systems. The work is based on the idea of directly adapting the concept of behaviour-oriented robustness to stochastic models. Individual simulated trajectories of the CTMC are locally analysed with respect to a formula of Signal Temporal Logic (STL), a linear-time temporal logic interpreted on simulated time sequences. For each simulated trajectory, the so-called satisfaction degree representing the distance from being (un)satisfied is computed, thus resulting into a randomly sampled distribution of the satisfaction degree. This distribution thus gives another source of information in addition to the probability of formula satisfaction (percentage of valid trajectories in the sampled set). In comparison, our method directly (and exactly) computes the probability of formula satisfaction for a different kind of temporal logic – the branching-time CSL logic. This allows to express more intricate properties that require branching time, e.g., multi-stability. On the other hand, our method is based on transient analysis, not allowing to compute the local analysis of individual trajectories, i.e., to obtain the satisfaction degree would require non-trivial elaboration at the level of numerical algorithms.

In the domain of ODE models, there exist several analytic methods for effective analysis under parameter uncertainty. They build on the static analysis (stoichiometric analysis, flux balance analysis) as well as dynamic numerical methods (simulation, monitoring by temporal formulae, sensitivity analysis) implemented in tools (e.g. [29]–[31]). Robustness analysis with respect to functionality specified in terms of temporal formulae has been recently introduced [32], [33]. There exist two major approaches of defining and analysing robustness. If only the parameters of the model are perturbed, we speak of a *behaviour-oriented approach* to robustness. This approach has been explored by Fainekos and Pappas [32], further extended by A. Donzé et al. [34] and implemented in the toolbox Breach [35]. Another option could be to perturb the model structure, i.e., the reaction topology, as done in gene knock-outs. Such changes are in principle discrete and the problem of robustness computation for such perturbations could be reduced to solving many instances of the same problem for each topology. However, identifying model behaviour shared among individual perturbations can lead to more efficient analysis [36].

Yet another way to look at perturbations is from the perspective of property uncertainty. If the system is considered fixed and all parameters exactly known, the uncertainty then lies in the property of interest. For a specific property such as “The concentration of X repeatedly rises above 10 and drops below 5 within the first 20 minutes”, where all three numerical constants can be altered, we explore how much would they have to be altered in order to affect the property’s validity in the given model. This approach has been adopted for ODEs by F. Fages et al. [33] and implemented in the tool BIOCHAM [31]. When only the parameters of the property are perturbed, it is the case of a *property-oriented approach* to robustness.

A specific emphasis has been also given to the analysis of stochastic noise in deterministic dynamical systems modelled in terms of ODE [10] or discrete-time difference equations [37]. The main motivation is the robust design of synthetic biological networks that can ensure robust implementation of desired features at the level of cell regulatory mechanisms and signalling pathways. The considered models do not incorporate stochasticity at the level of molecular dynamics but only at the level of parameter uncertainty. The approach allows to analyse robustness of stochastically fluctuating parameters analytically. However, the stochasticity of biochemical interactions is completely neglected, which implies that those methods do not consider intrinsic molecular noise. On the contrary, the method presented in this paper inherently and rigorously targets parameter uncertainty in stochastic dynamics. As shown in the second case study, the results give us detailed insights into robustness of stochastic dynamics. Such a level of detail cannot be achieved with deterministic models.

## Methods

### Methodology Overview

**Figure 1:**
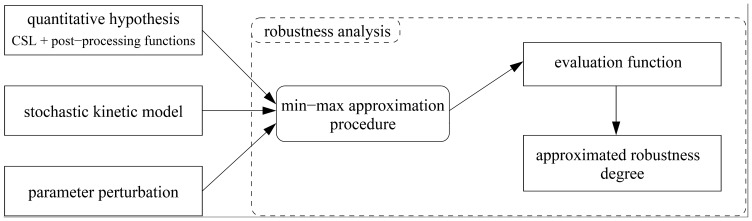
Robustness analysis workflow. The robustness analysis framework considers several objects on the input side. In particular, stochastic kinetic model is supplied with the quantitative hypothesis and the perturbation space of interest. The robustness analysis procedure systematically traverses the perturbation space and explores the system’s functionality determined by the quantitative hypothesis. The output side of the framework provides the evaluation function describing the system’s functionality with respect to the perturbation space. A single value characterising the system robustness is computed by integrating the evaluation function over the perturbation space.

The framework for robustness analysis implements a workflow that is briefly summarised in Figure 1. The following objects make the input of the workflow:

- **Stochastic kinetic model**. A finite state model (with semantics given by CTMC) defined by a set of chemical species participating in a set of chemical reactions (each species has a bound specifying its maximal population)
- **Parameter perturbation.** A perturbation space defined by a Cartesian product of uncertain stochastic rate constants (given as intervals with minimal and maximal values) and initial conditions of the system (given as a set of states representing initial populations of particular species)
- **Quantitative hypothesis about the system.** Stochastic temporal property formalised using the bounded time fragment of CSL extended with rewards and post-processing functions that is interpreted over the paths and states of CTMC.

The procedure of *robustness analysis* considers the given CTMC 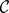 that is explored with respect to the CSL formula 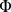 over the space of perturbations 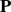. The perturbation space can be discrete but still very large or continuous and thus infinite. The central goal of the procedure is to efficiently approximate the evaluation function 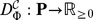, which for each parameter point (parameterisation) 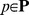 returns the quantitative model checking result for the respective CTMC 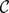 (built for the parameterisation 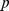) and the given property 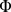. Depending on the property 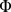, the value 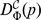 represents the probability, the expected reward or the value of a post-processing function corresponding to the parameterisation 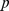.

The approximation of the evaluation function 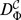 is the main output of the framework. It is further processed in order to obtain a single aggregated value that characterises the *robustness degree* of the model with respect to the perturbations 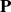 and the property 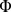. To effectively approximate the function 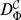, we employ the min-max approximation method recently published in [8]. The method guarantees upper and lower bounds of the function 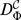 without neglecting any sharp changes or discontinuities. This method exploits numerical techniques for probabilistic model checking, can provide arbitrary degree of precision, and thus can be considered as an orthogonal approach to the parameter sampling and adaptive grid refinement embedded within statistical techniques.

The framework extends the min-max approximation to a more general class of stochastic biochemical models (i.e., incorporation of stochastic Hill kinetics) and a more general class of quantitative properties (i.e., including post-processing functions), and allows us to compute the robustness degree of such systems. In our framework we provide the user not only a single value characterising the robustness of the system but also the landscape visualisation of the evaluation function.

In the next subsections, we describe all the components of the framework in detail.

### Model

The formalism used to model a biochemical system is essential, since it not only dictates the possible behaviours that may or may not be captured, but also determines the means of detecting them. ODEs enable the study of large ensembles of molecules in a population, since they abstract from individualistic properties of each molecule, such as position or its stochastic behaviour, and take only concentrations of each species as its variables. Stochastic models such as CTMCs abstract from positions of molecules but maintain their individual interactions. Even more detailed models such as Brownian dynamics, which keep track of positions but abstract from the geometry and orientation of each molecule, could be used. However, as the amount of information about each individual molecule increases, the computational complexity of proving that some property holds over all behaviours of a model becomes quickly infeasible even for small models.

In our framework we focus on stochastic biochemical systems that can be formalised as a finite state system 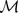 defined by a set of *N chemical species* in a well-stirred volume with fixed size and fixed temperature participating in *M chemical reactions*. The number 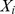 of molecules of each species 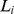 has a specific bound and each reaction is of the form 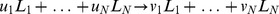 where 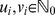 represent *stoichiometric coefficients*.

A *state* of a system in time 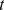 is the vector 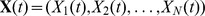. When a single reaction with index 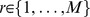 with vectors of stoichiometric coefficients 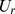 and 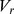 occurs the state changes from 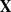 to 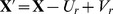, which we denote as 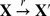. For such reaction to happen in a state 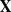 all reactants have to be in sufficient numbers and the state 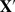 must preserve all species bounds. The *reachable state space* of 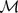, denoted as 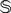, is the set of all states reachable by a finite sequence of reactions from *an initial state* 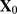. The set of indices of all reactions changing the state 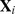 to the state 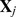 is denoted as 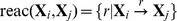. Henceforward, reactions will be referred directly by their indices.

According to [5], [13] the behaviour of a stochastic system 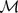 can be described by the CTMC 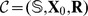 where the transition matrix 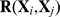 gives the probability of a transition from 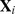 to 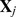. Formally, the transition matrix 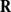 is defined as:
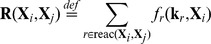
where 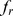 is a *stochastic rate function* and 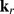 is a vector of all numerical parameters occurring in 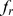 such as a *stochastic rate constant* 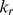, stoichiometry exponents, Hill coefficients etc.

In the case of mass action kinetics, the stochastic rate function has the simple form of a polynomial of reacting species populations. That is 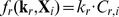 where 
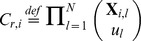
 corresponds to the population dependent term such that 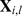 is the *l*th component of the state 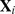 and 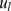 is the stoichiometric coefficient of the reactant 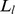 in reaction 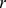. However, sometimes the mass action kinetics is not sufficient, especially, when the reactions are not elementary but rather form an abstraction of several reactions with unknown precise topology (e.g., gene transcription) or if including all elementary reactions would cause the analysis to be computationally infeasible. In such cases, the dynamics is typically approximated by Hill functions [38], a quasi-steady-state approximation [39] of the law of mass conservation. For the sake of simplicity, we will further assume that for each reaction 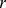 the vector 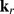 is one-dimensional and thus 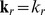, the proposed methods can however be directly used also for multi-dimensional vectors of constants. To comply with the standard CTMC notation, states 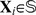 will be henceforward denoted as 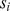.

The probability of a transition from state 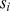 to 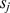 occurring within *t* time units is 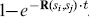, if such a transition cannot occur then 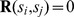. The time before any transition from 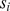 occurs is exponentially distributed with an overall *exit rate* 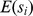 defined as 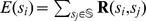. A path 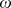 of CTMC 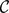 is a non-empty sequence 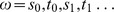 where 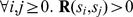 and 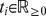 is the amount of time spent in the state 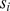 for all 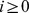. For all 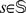 we denote by 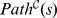 the set of all paths of 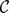 starting in state 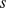. There exists the unique probability measure on 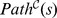 defined, e.g., in [40]. Intuitively, any subset of 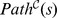 has a unique probability that can be effectively computed. For the CTMC 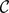 the transient state distribution 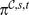 gives for all states 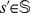 the transient probability 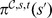 defined as the probability, of being in state 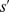 at the finite time *t*, having started in the state *s*.

### Perturbations

In our approach we have focused on the behaviour-oriented approach to the robustness of stochastic systems and thus we will now define a set of perturbed stochastic systems and their CTMCs. Let each stochastic rate constant 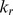 have a value interval 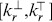 with minimal and maximal bounds expressing an *uncertainty range* or *variance* of its value. A *perturbation space* 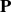 induced by a set of stochastic rate constants 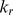 is defined as the Cartesian product of the individual value intervals 
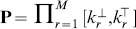
. A single *perturbation point* 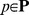 is an *M*-tuple holding a single value of each rate constant, i.e., 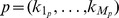.

A stochastic system 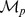 with its stochastic rate constants set to the point 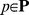 is represented by a CTMC 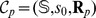, where the transition matrix 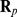 is defined as:
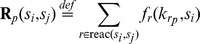



A *set of parameterised* CTMCs induced by the perturbation space 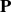 is defined as 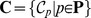.

Additionally, we consider the perturbation of initial conditions of the stochastic system that are represented by different initial states of the corresponding CTMC. In this case we extend the perturbation space such that a single perturbation point 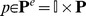 where 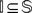 is an *M*+1-tuple holding a single value of an initial state and a single value of each rate constant, i.e., 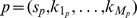 and CTMC 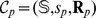.

### Functionality

To be able to automatically analyse a system’s function 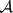 under scrutiny there must be a formal way of expressing 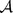. A function of a system in the biological sense is any intuitively understandable behaviour such as response, homoeostasis, reproduction, respiration, or growth. It can be a high level concept such as chemotaxis as well as a low level one, e.g., reaching a state with a given number of molecules of a specific species.

The inspected function can usually be described by a *property* that is understood as an abstraction of a system’s behaviour expressed in some temporal logic and given as a formula of that logic. Unlike the intuitive concept of a biological function mentioned above, a property may be formally verified over a formal model of a system and proven to hold or to be violated. Since the concept of robustness builds on the notion of a function that can be measured, we focus on a quantitative logic for stochastic systems. We use *continuous stochastic logic* (CSL) [6], [41] extended with *reward* operators [7]. In our framework we focus only on the *bounded time fragment of CSL* that allows us to speak only about behaviour within a finite time horizon. For most cases of biochemical stochastic systems, such as intracellular reaction cascades or multi-cellular signalling, the bounded time restriction is adequate since a typical behaviour is recognisable within finite time intervals [42].

Formal syntax and semantics of the bounded time fragment of CSL with rewards are briefly presented in Text S1 (Section I). Intuitively, a CSL formula consists of *temporal operators* allowing to reason about path propositions qualified in terms of time, and *probabilistic operators* allowing to quantify required probability thresholds for particular path propositions. *Reward operators* introduce cost functions that enable to express properties such as the probability of a system being in the specified set of states over a time interval or the probability that a particular reaction has occurred. Generally, reward operators allow to express properties specifying the expected value of an expression defined using the cost functions.

There exist biologically relevant properties that cannot be directly expressed using reward operators. As an example we can mention the property that is analysed in the second case study, i.e., degree of the population noise given by a *mean quadratic deviation* (*mqd*) of the population probability distribution of a species at a given time. Reward operators cannot be used in this case since they require *a priori* known cost functions. Therefore, we employ a class of *post-processing* functions to further broaden the scope of behaviour that can be formally captured. The key idea is to replace a cost function by a post-processing function that aggregates the transient state distribution at the given finite time. A formal concept of CSL with post-processing functions is also presented in Text S1.

**Figure 2:**
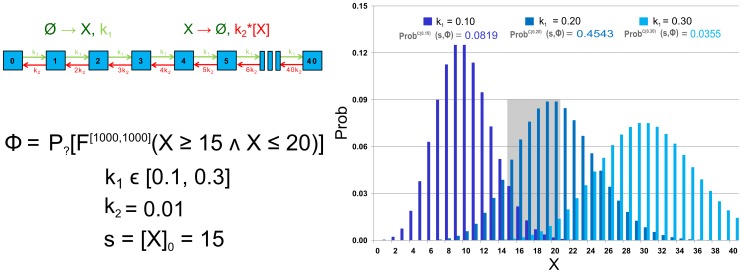
Running example. The example model contains one species *X* with the population bounded to 40, two reactions: production of *X* (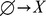 with rate 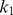), degradation of *X* (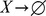 with the rate 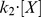, 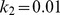) and the initial population of 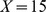. The corresponding CTMC has 41 states (initial state 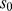 corresponds to the state with initial population). The inspected formula 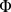 represents the quantitative property “What is the probability that the population of 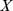 is between 15 and 20 at time 1000?” The perturbation space 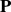 is given by the interval of the stochastic rate constant 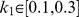. On the right, there are depicted three transient distributions at time 1000 for three different values of 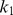 and the resulting probabilities for the formula 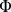 obtained as the sum of probabilities in states with populations from 15 to 20.

To demonstrate that the bounded time fragment of CSL with rewards and post-processing functions can adequately capture relevant biological behaviour and thus can be successfully used in the robustness analysis of stochastic biochemical systems, we list several formalisations of such behaviours:

- **Stochastic reachability.** 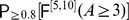 expresses the qualitative property “The probability that the population of *A* reaches 3 between 5 and 10 time units is at least 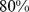
”. Another example of a stochastic reachability property is shown in Figure 2.
- **Stochastic stability.** 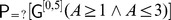 represents the quantitative property “What is the probability that the population of *A* remains between 1 and 3 during the first 5 time units?”
- **Stochastic temporal ordering of events.** 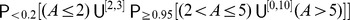 expresses the qualitative stochastic version of the following temporal pattern: “Species A is initially kept below 2 until it reaches 5 and finally exceeds 5.” The formula quantifies both the time constrains of the events and the probability that the events occur. It expresses that ’’The probability that the system has the following probabilistic temporal pattern is less than 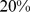: the population of *A* is initially kept below 2 until the system between 2 and 3 time units reaches the states satisfying the subformula 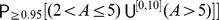. “The subformula specifies the states where ’’The probability that the population of *A* remains greater than 2 and less or equal 5 until it exceeds 5 within 10 time units, is greater than 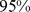.”
- **Cumulative reward property.** 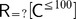, where 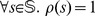 iff 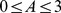 in *s*, captures the quantitative property “What is the overall time spent in states with population of *A* between 0 and 3 within the first 100 time units”, which can also be understood as “What is the probability of the system being in a state with population of *A* between 0 and 3 within the first 100 time units”.
- **Noise as mean quadratic deviation.** 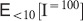, where the post-processing function is defined as 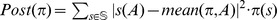, 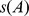 gives the population of *A* in state *s* and 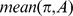 is the mean of the distribution 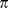 defined as 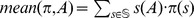. This qualitative property states that “The mean quadratic deviation of the distribution of species *A* at time instant 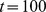 must be less than 10”.

We say that a formula 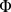 has *qualitative* semantics if the topmost operator of 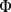 specifies a threshold 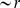 (e.g., a qualitative property 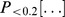), and *quantitative* semantics, if the threshold is not specified (e.g., quantitative property 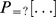). For a given CTMC 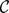 and a CSL formula 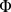 with the qualitative semantic, the result of the *model-checking procedure* has the form of a boolean yes/no answer. If 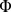 has the qualitative semantics, the result has the form of a numerical value corresponding to the probability, the expected reward, or the post-processing function. As we will show the quantitative semantics is more suitable for robustness analysis.

An example including a simple one-dimensional model with two reactions (production and degradation), its CTMC representation, and a quantitative CSL formula, is depicted in Figure 2. The perturbation space of the model is given by the interval of the production rate. Figure 2 also depicts three transient distributions for three different values of the production rate and the resulting probabilities for the formula.

### Robustness Degree

Let us recall the general definition of robustness as given by Kitano [2] to make more explicit its possible intepretations and also to show how we propose to use it in the context of stochastic systems.
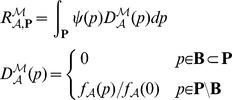



### Functionality Evaluation

Kitano proposed that the evaluation function 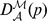, stating how much the functionality 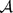 is preserved in perturbation *p*, should be defined using a subspace 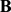 of all perturbations, where the system’s function is completely missing and the remaining 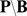 where the function’s viability is somehow altered. This definition is meaningful, e.g., in cases where the perturbation would lead to a system not having the considered function at all (speed of reproduction of a dead cell) or in cases where a plain measurement would provide a function’s value, though in reality the system would lack the function (inside temperature during homoeostasis experiment in conditions when an organism loses thermal control and has temperature of the environment). These examples have in common that the information about a system lacking its function is provided from outside because if it could be deducible from the system’s state alone, it could be incorporated into the evaluation function 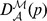 itself.

For perturbations 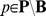 where the system maintains its function at least partially, Kitano proposes to express the evaluation function 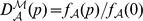 relatively to the ground (unperturbed) state 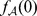. This is meaningful, e.g., for naturally living systems where the ground state is measurable and is considered as an optimal performance state. Such a definition enables the comparison of some common property of different species. For example, the reproduction rate for a mouse and a sequoia tree with respect to perturbations of their environment. If a mouse has 20 offsprings per year in the base temperature and 22 offsprings for a 2 Kelvin rise, then the evaluation function 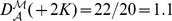. While if a sequoia has 1000 seedlings in the ground temperature and1200 for the 2 Kelvin rise then 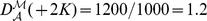.

The relativistic nature of Kitano’s definition allows to compare robustness of otherwise incomparable organisms. In our example, the sequoia is more robust to the single perturbation of temperature by 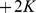 than the considered species of mice. Onthe other hand, in cases when no ground state is given, the absolute value can provide more adequate measure of robustness.

In the next section we propose several different definitions of robustness in stochastic systems providing both the *absolute* and the *relative* interpretations.

### Robustness in Stochastic Systems

Consider a stochastic system described by a CTMC 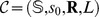, perturbation space 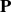 and CSL formula 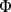 formalising the system’s function 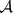. Notice that the CTMC 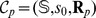 represents the system with stochastic rate constants set to the point 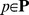. In cases where the perturbation space 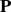 is extended by initial conditions (i.e., a single perturbation point 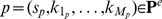), the corresponding CTMC is defined as 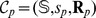.

Let 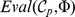 be an auxiliary function (formally defined in Section III in Text S1 ) that returns the numerical value representing the quantitative model-checking result for the CTMC 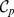 and the formula 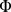. It means, that the possible threshold 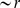 (where 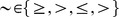) in the top most operator of 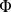 is ignored (i.e., it is treated as 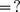). Given these specifications the evaluation function 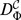 can be restated in several different ways:
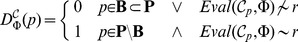
(1a)




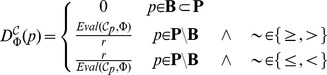
(1b)




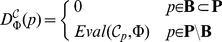
(1c)




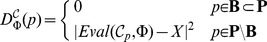
(1d)where 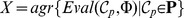 and 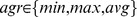. The degree of robustness, further denoted as 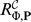, can be now defined as the integral of the evaluation function 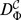 over the perturbation space 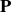:



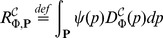
where 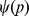 is the probability of the perturbation 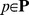.

The first two definitions are possible for specifications where the topmost operator of the formula 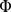 includes the threshold 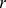. In the first definition (1a) the evaluation function 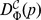 returns a qualitative result, therefore robustness 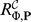 specifies the measure of all perturbations in 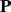 for which the property holds in a strictly boolean sense – it is the fraction of 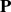 where the property is valid. This definition can be used, e.g., in the property 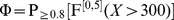, which specifies that in 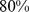 of cases the population of X increases above 300 within 5 seconds. For this property and a model with a parameter 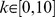 the robustness gives us the fraction of the parametric interval 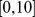 for which the model satisfies 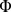.

In the second definition (1b) the evaluation function 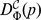 returns the quantitative value that is relative to threshold *r*. Therefore, robustness can be interpreted as the average relative validity of the property over 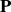. If *r* corresponds to the validity of 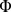 in conditions considered natural for the inspected system 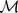 (i.e., to the unperturbed state) then this interpretation complies with the original definition of Kitano. Let us consider the same property 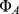 and the same parametric space 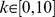. If for all values of *k* the model has a 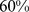 change that its behaviour will lead to a population of X larger than 300 within 5 seconds than its robustness is 0.6/0.8 = 0.75. If the probability is different in each *k* then robustness gives us the average value with which our expectations will be met.

The third definition (1c) is possible for specifications using the quantitative semantics of formula 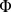. Here robustness gives the mean validity over all 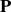, regardless of any probability threshold *r*. This interpretation is convenient when there are no *a priori* assumptions about the system’s expected behaviour.

Finally, to express the fact that the system behaviour remains the same (with respect to the evaluation function) across the space of perturbations, we introduce the fourth definition (1d). It uses an aggregation function to compute a mean value and expresses the variance from the mean. This definition enables us to compare models which have the same numerical values of robustness in the sense of definition (1c) but which achieve the average value with very different landscapes of evaluation function.

While the last three definitions require precise computation of the probability value in every 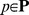, the first definition is amenable to approximate solutions. In this case it suffices to ensure that the probability is larger or smaller than *r*. In many cases it can be achieved without computing the precise value and thus statistical model checking techniques can be used efficiently. In both case studies, we use definition (1c), since we do not consider any ground unperturbed state. We assume 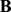 to be an empty set and expect the lack of functionality 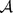 to be fully expressible in terms of the property 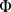.

### Robustness Analysis Procedure

Having the definition of the evaluation function 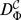 we can describe an effective method for computation of the robustness degree 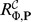. Let us first consider the case where the perturbation space 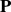 does not contain different initial states.

The evaluation of 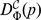 includes the computation of 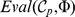, i.e., the solution of standard CSL model checking problem. Since the problem can be rather complex even for a single perturbation point 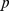, an explicit computation of the integral over the whole space of perturbations is infeasible. Therefore, we consider an approximation of the evaluation function 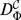 using the upper bound 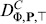 and the lower bound 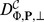 with respect to 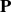 defined as:
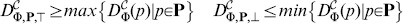
(2)


This approximation is in most cases too course and thus we use a finite decomposition of the perturbation space 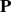 into perturbation subspaces 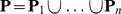. This approach allows to effectively compute the upper bound 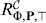 and lower bound 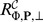 of the robustness degree 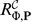 in the following way:
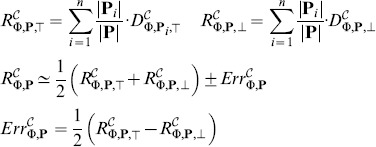
(3)


Let us now consider the case in which the perturbation space is extended with initial states, i.e., 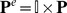 where 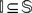 and 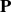 is non-singular. For this case the integral defining robustness is actually a finite sum of integrals:
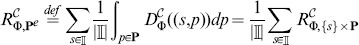
(4)


Equations 3 and 4 valid only for a uniform distribution of the perturbation probability 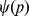 over the whole space of perturbations 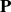 and 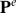, respectively. However, our approach can be straightforwardly modified for non-uniform distributions.

Using (4), the robustness computation for perturbations containing a single initial state can be easily extended to perturbations containing different initial states. In Text S1 (Section III) we show that for properties specified without post-processing function the global model checking procedure (utilised in the robustness computation) returns results for an arbitrary set of initial states 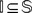 with the same time complexity as for a single state.

As we can see, the key step in our approach is to compute the values 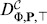 and 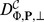 for the given CTMC 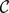, the formula 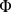 and the perturbation space 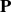. In our framework we extend our previous method called *min-max approximation* [8], thus allowing to effectively approximate the evaluation function 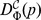. Intuitively, for a formula 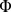 the min-max approximation computes the upper and lower bounds of the function 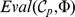 with respect to all perturbation points 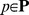. Afterwards, these bounds are used to obtain the values 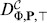 and 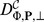 such that Equation 2 is satisfied.

Similarly as the standard CSL model-checking methods [40], [43], the min-max approximation reduces the model-checking problem of a set of parameterised CTMCs to the computation of the upper and lower bounds of a transient probability distribution in a finite time. Remark that this reduction can be used only for the time bounded fragment of CSL, which is our case. The key idea of the min-max approximation is to replace the uniformisation (the standard technique for transient analysis) by a novel technique called *parameterised uniformisation* [8].

### Parameterised Uniformisation and Min-Max Approximation

The parameterised uniformisation is a novel modification of the standard uniformisation [40], a widely used technique for transient analysis of CTMCs (see Section II in Text S1 for more details). For the given set of parameterised CTMCs 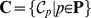, the initial state 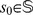 and time 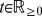, the parameterised uniformisation returns vectors 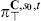 and 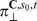, such that for each state 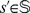 the following holds:
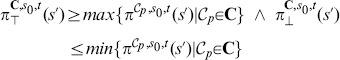
where 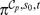 denotes the transient state distribution of CTMC 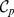 in the time 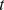. The key idea of the modification is to compute for each state 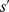 the local maximum (minimum) of 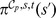 over all 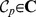 with respect the current computation step of 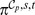. It means that only the maximal (minimal) values of predecessors of 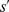 from the preceding step are considered. To obtain the local maximum (minimum) of 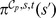 we define a function returning for the perturbation point 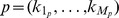 the difference of probability mass inflow and outflow to/from state *s*. In [8] we have shown that if all reactions are described by mass action kinetics the function is monotonic with respect to any single perturbed stochastic rate parameter 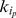. This allows us to efficiently identify 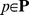 that maximises (minimises) the value 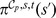 and thus to obtain the vectors 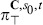 and 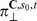.

For more complex rate functions than those resulting from mass action kinetics, the corresponding function does not have to be in general monotonic over 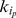 for all states *s*. This makes the computation of local extremes more intricate, though still tractable. In Text S1 (Section V) we describe a novel extension of the parametric uniformisation allowing to analyse models with more complex rate functions.

The aforementioned parameterised uniformisation can be straightforwardly employed also for backward transient analysis that is used for the global model checking procedure. For the given set of states 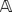 and time 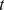 we can efficiently compute the vectors 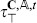 and 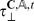 such that for each state 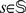 the following holds:
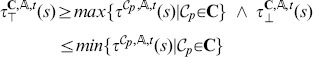
where 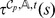 denotes the probability that the set 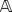 is reached from 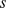 at time *t* in the CTMC 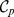.

The min-max approximation employs the results of the parameterised uniformisation (i.e., the vectors 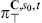, 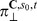 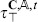 and 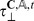) to approximate the *largest set of states satisfying* 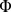, and the *smallest set of states satisfying* 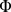 with respect to the space of perturbations 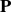. It computes the approximation 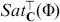 and 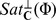 such that
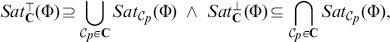
where 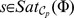 iff 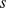 satisfies the formula 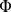 in CTMC 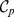. To obtain such approximations we extended the standard satisfaction relation for CSL logic [8]. The sets 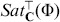 and 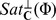 are further used to compute the values 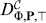 and 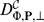. For more details about the min-max approximation see Text S1 (Section IV) and [8].

For a general class of post-processing functions the results of the parameterised uniformisation cannot be directly used to compute the values of 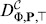 and 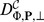 that would satisfy Equation 5, since there is no guarantee about the projective properties of the functions. Therefore, in Text S1 (Section V) we show how the min-max approximation method can be extended for the post-processing function defined as the mean quadratic deviation of a probability distribution. This extension allows us to quantify and analyse the noise in different variants of signalling pathways that are studied in the second case study.

### Accuracy of Min-Max Approximation and Perturbation Space Decomposition

Our approach introduces an *overall error*, denoted as 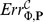, given as
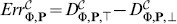



The overall error is composed of two parts: 1) the inaccuracy related to the min-max approximation of the evaluation function, called *approximation error* and 2) the inaccuracy related to the parameterised uniformisation, called *uniformisation error*. The approximation error is given as
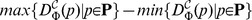



The unformisation error is caused by the fact that the parameterised uniformisation in general does not correspond to standard uniformisation for any CTMC 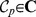. The reason is that we consider a behaviour of a parameterised CTMC that has no equivalent counterpart in any particular 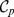. First, the parameters (minimising/maximising the inspected value) are determined locally and thus independently for each state. Second, the parameters are determined independently for each computational step. The uniformisation error is given as
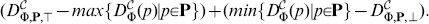



**Figure 3:**
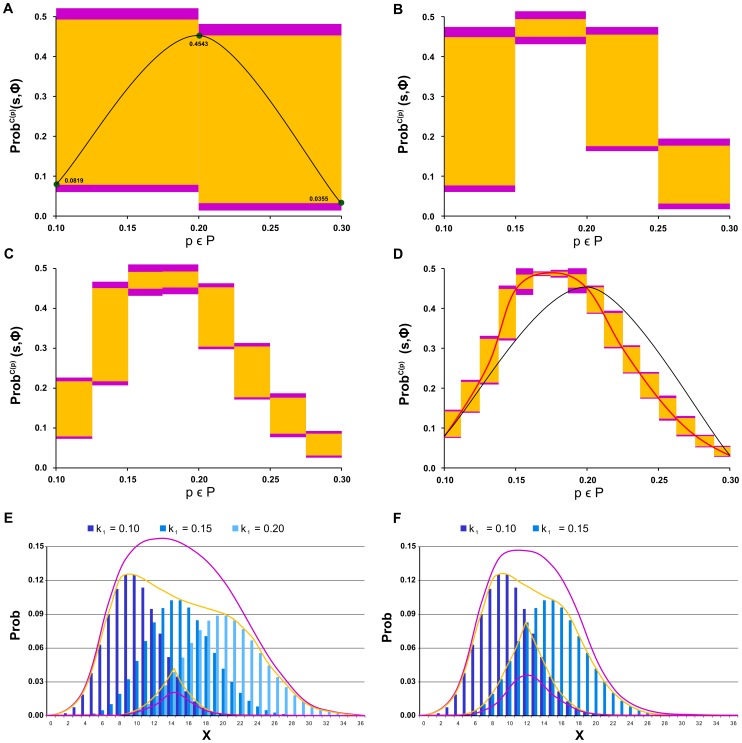
Perturbation space refinement. Part (A) depicts three resulting probabilities (green dots) of the formula 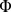 (for the initial state 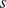), denoted as 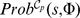, for three values of the rate constant 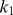 corresponding to three perturbation points 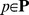 from Figure 2. The shape of 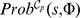 for all 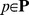 is estimated at these three points by polynomial interpolation and shown as a black curve. The top four parts (A), (B), (C) and (D) illustrate the min-max approximation of the evaluation function (i.e., the values 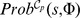 for all 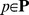) using the decomposition of 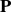 into 2, 4, 8 and 16 subspaces. The exact shape of the evaluation function is visualised as the red thick curve in (D) and is compared to the initial estimate and to the min-max approximation. Two types of errors are illustrated: the approximation error is depicted as yellow rectangles and the uniformization error as the purple rectangles. As can be seen, a more refined decomposition reduces both types of errors in each further refined subspace. Part (E) depicts how the errors arise during the computation of parametrised uniformisation. The yellow curves illustrate the minimal and maximal transient probability distributions with respect to the inspected interval of the parameter 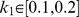. The purple curves illustrate the approximations of the the minimal and maximal distributions computed using parametrised uniformisation. Part (F) demonstrates how the error can be reduced using perturbation space decomposition. It illustrates the errors for the parameter 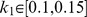.

Naturally, the overall error is equal to the sum of both errors. Figure 3 illustrates both types of errors, where the approximation and unification errors are depicted as the yellow and purple rectangles, respectively.

We are not able to effectively distinguish the proportion of the approximation error and the unification error nor to reduce the unification error as such. Therefore, we employ a finite decomposition of 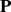 into perturbation subspaces in order to refine the min-max approximation of the evaluation function 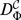 over the perturbation space 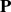. Our aim is to effectively reduce the overall error 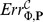 to a user-specified *absolute error bound*, denoted as 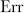. We iteratively decompose the perturbation space 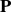, such that 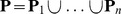 and each partial result satisfies the overall error bound, i.e., 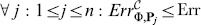. Therefore, the overall error equals to
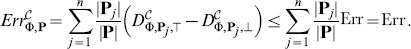



Figure 3 illustrates such a decomposition and demonstrates convergence of the overall error 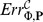 to 0, provided that the evaluation function 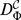 over 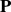 is continuous. If the function is not continuous and the discontinuity causes that the absolute error bound cannot be achieved, a supplementary termination criterion is applied. We provide a detail description of our decomposition strategy with respect to the user specified absolute error bound in Text S1 (Section VI).

**Figure 4:**
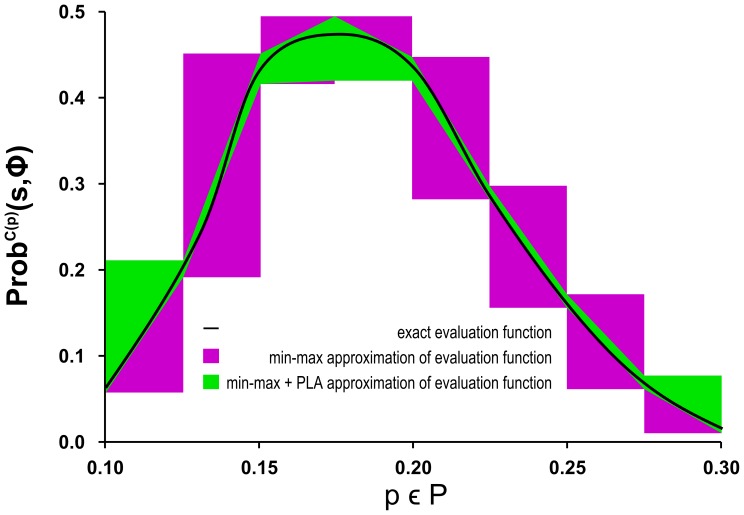
Piece-wise linear approximation. A piece-wise linear approximation (PLA) is shown in green. It is computed by linearly interpolating the grid points in which the upper and lower bounds of the evaluation function may be computed more precisely as the minimum resp. maximum of the values from all parameter subintervals sharing boundary grid points. The obtained result is more precise than the original min-max approximation (in purple), albeit without the conservative guarantee on bounds.

The accuracy of the approximation can be further improved using the *piece-wise linear approximation* (PLA). This concept is illustrated in Figure 4. Since the spaces 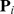 and 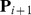 have a common point *p* (in a general *n* dimensional perturbation space 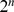 subspaces intersect in a single point *p*), we can use this to obtain a more precise range of values for the value of the property 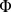 in *p* as
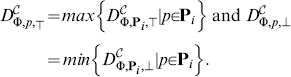



Under the assumption that the value of a property does not change rapidly over sufficiently small subspaces 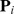, the resulting upper and lower bound can be computed from linear interpolation of grid points *p*. The decision in which cases is such an assumption acceptable is up to user, since there is in general no efficient way of resolving this situation. In such a case the overall piece-wise linear approximation will usually have a higher precision albeit without the guarantee of upper and lower bounds.

### Implementation

We delivered a prototype implementation of the framework for the robustness analysis on top of the tool PRISM 4.0 [44]. This tool provides an appropriate modelling and specification language. Our implementation builds on the sparse engine that uses data structures based on sparse matrices. They provide suitable representation of models for time-efficient numerical computation.

In the case that a large number of perturbation subspaces is required to obtain the desired accuracy of the approximation, the sequential computation can be extremely time consuming. However, our framework allows very efficient parallelisation, since the computation of particular subspaces is independent and thus can be executed in parallel. Therefore, the robustness analysis can be significantly accelerated using high-performance parallel hardware architectures.

## Results

### Gene Regulation of Mammalian Cell Cycle

**Figure 5:**
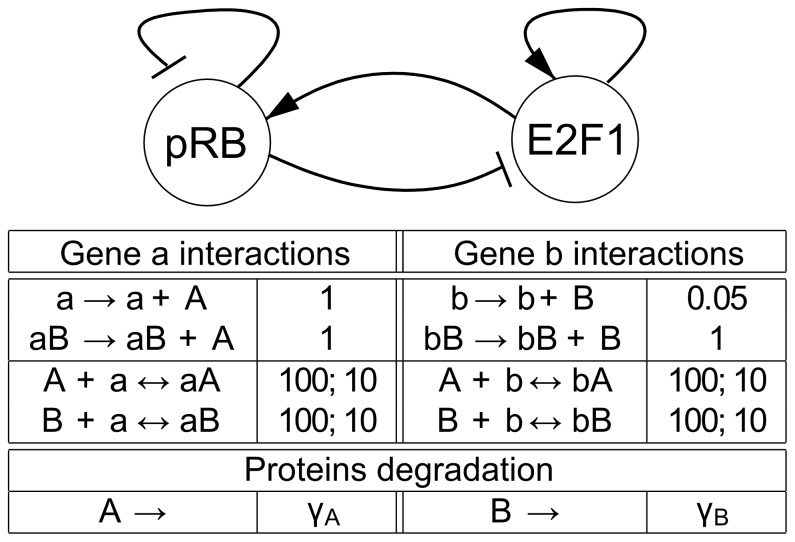
Model of regulation of the mammalian cell cycle. The core gene regulatory module controlling the 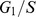-phase transition in the cell cycle of mammalian cells [45] is depicted in the upper part. The retinoblastoma protein *pRB* (A) [HumanCyc:HS06650] interacts with the retinoblastoma-binding transcription factor 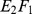 (B) [HumanCyc:HS02261]. In high concentration levels, the 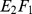 protein activates the 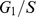 transition mechanism. On the other hand, low concentration of 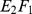 prevents committing to 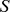-phase. Positive autoregulation of 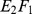 causes bistability. Stochastic mass action reformulation of the 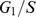 regulatory circuit is shown in the table below. The gene regulation is modelled by means of a set of second-order reactions simplifying the elementary processes behind transcription. In particular, the model includes the interactions among transcription factors (*A*, *B* stand for *pRB* and 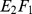, respectively) and respective genes and protein production/degradation reactions. The interactions are represented by reversible TF-gene binding reactions in the second row of the table (genes are denoted by small letters). Individual protein production reactions controlled by these interactions are represented by the irreversible gene expression reactions in the first row of the table. Protein degradation is modelled as spontaneous by means of first-order reactions. Kinetic coefficients are set only approximately provided that they are considered equal for all instances of a particular process (binding, dissociation, promoted protein production). The only exception is the spontaneous (basal) expression of *b* which is set to a low rate. This mimics the fact that 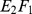 is only rapidly produced under the circumstances of self-activation [9]. Degradation parameters are left unspecified.

We have applied the robustness analysis to the gene regulation model published in [45], the regulatory network is shown in Figure 5 (left). The model explains regulation of a transition between early phases of the mammalian cell cycle. In particular, it targets the transition from the control 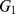-phase to *S*-phase (the synthesis phase). 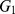-phase makes an important checkpoint controlled by a *bistable regulatory circuit* based on an interplay of the retinoblastoma protein *pRB*, denoted by *A* (the so-called tumour suppressor, HumanCyc:HS06650) and the retinoblastoma-binding transcription factor 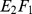, denoted by *B* (a central regulator of a large set of human genes, HumanCyc:HS02261). In high concentration levels, the 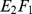 protein activates the 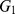/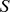 transition mechanism. On the other hand, a low concentration of 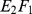 prevents committing to *S*-phase.

Positive autoregulation of *B* causes bi-stability of its concentration depending on the parameters. Of special interest is the degradation rate of *A*, 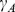. In [9] it is shown that for increasing 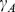 the low stable mode of *B* switches to the high stable mode. When mitogenic stimulation increases under conditions of active growth, rapid phosphorylation of *A* starts and makes the degradation of unphosphorylated *A* stronger (the degradation rate 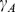 increases). This causes *B* to lock in the high stable mode implying the cell cycle commits to *S*-phase. Since mitogenic stimulation influences the degradation rate of *A*, our goal is to study the population distribution around the low and high steady state and to explore the effect of 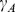 by means of the evaluation function.

It is necessary to note that the original ODE model in [9] has been formalised by means of Hill kinetics representing the cooperative action of transcription factor molecules. Since Hill kinetics cannot be directly transferred to stochastic modelling [46], [47], we have reformulated the model in the framework of stochastic mass action kinetics [5]. The resulting reactions are shown in Figure 5 (right). Since the detailed knowledge of elementary chemical reactions occurring in the process of transcription and translation is incomplete, we use the simplified form as suggested in [4]. In the minimalist setting, the reformulation requires addition of rate parameters describing the transcription factor-gene promoter interaction while neglecting cooperativeness of transcription factors activity. Our parameterisation is based on time-scale orders known for the individual processes [48] (parameters considered in 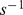). Moreover, we assume the numbers of *A* and *B* are bounded by 10 molecules. Correctness of the upper bounds for *A* and *B* was validated by observing a thousand independent stochastic simulations. We consider minimal population number distinguishing the two stable modes. All other species are bounded by the initial number of DNA molecules (genes *a* and *b*), which is conserved and set to 1. The corresponding CTMC has 1078 states and 5919 transitions.

We consider two hypotheses: (1) stabilisation in the low mode with 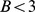, (2) stabilisation in the high mode with 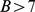. Both hypotheses are expressed within the time horizon of 1000 seconds, reflecting the time scale of gene regulation response. According to [9], we consider the perturbation space 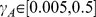. For both hypotheses we consider three different settings of 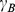: 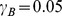, 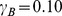, and 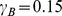.

**Figure 6:**
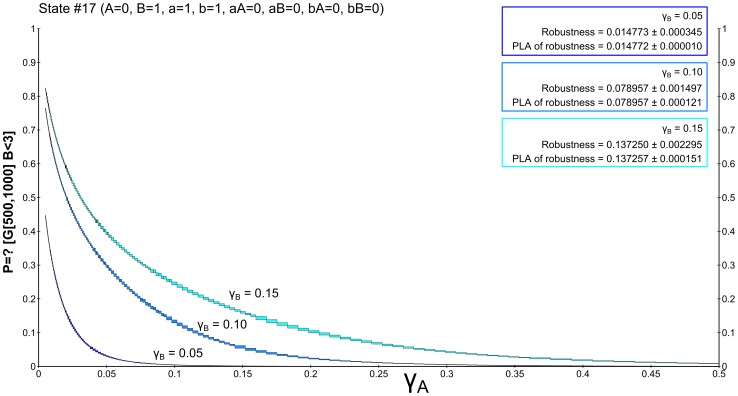
Results of robustness analysis for hypothesis (1) using the until operator. Hypothesis (1) requires stabilisation of 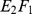 in the low concentration mode (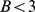). A CSL formula with the until operator is used in this case. Each of the curves represents the evaluation function over 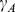 degradation obtained for a particular setting of 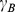. More precisely, the horizontal axis shows the perturbation of *pRB* degradation rate and the vertical axis shows the probability of the hypothesis to be satisfied. In the upper left corner, robustness values are shown for each of the curves. The values are displayed with the absolute error quantifying the precision of the approximate method. For comparison, the values are computed also on piece-wise affine approximations of the evaluation function. It can be seen that the robustness values are small which is due to the fact that fluctuations of molecular numbers cause frequent crossing of the required bound in the considered time horizon.

We employ two alternative CSL formulations to express the hypothesis (1). First, we express the property of being inside the given bound during the time interval 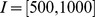 using the globally operator: 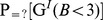. The interval starts from 500 seconds in order to avoid the initial fluctuation region and let the system stabilise. The resulting landscape visualisation is depicted in Figure 6, together with the robustness values computed for individual cases. Since the stochastic noise causes molecules to repeatedly escape the requested bound, the resulting probability is significantly lower than expected. Namely, in the case 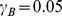, the resulting probability is close to 0 for almost all considered parameter values implying very small robustness. Increasing the *B* degradation rate causes an observable increase in robustness.

**Figure 7:**
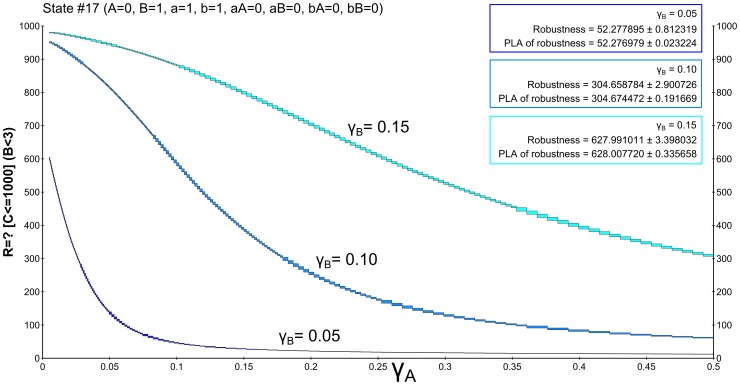
Results of robustness analysis for hypothesis (1) using the reward operator. Hypothesis (1) requires stabilisation of 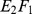 in the low concentration mode (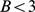). A CSL formula with cumulative reward operator is used in this case. Each of the curves represents the evaluation function over 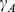 degradation obtained for a particular setting of 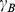. More precisely, the horizontal axis shows the perturbation of *pRB* degradation rate and the vertical axis shows the probability of the hypothesis to be satisfied. In the upper left corner, robustness values are shown for each of the curves. The values are displayed with the absolute error quantifying the precision of the approximate method. For comparison, the values are computed also on piece-wise affine approximations of the evaluation function. It can be seen that the robustness values change rapidly with different settings of 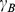. This observation goes with the fact that with faster degradation of 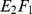 there is a higher probability that the positively self-regulated protein is locked in the stable mode of no production. The decrease of the value with increasing 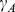 is due to the weakening effect of inhibition by *pRB*.

In order to prevent fluctuations from affecting the result, we use a cumulative reward property to capture the fraction of time the system has the required number of molecules within the time interval 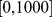: 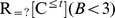, where 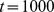 and 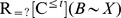 denotes that state reward 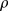 is defined such that 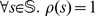 iff 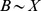 in 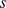. The resulting landscape visualisation is shown in Figure 7. Here the effect on the increase of robustness value with respect to increasing 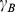 is significantly stronger.

After normalising the robustness values, we can observe that the model is significantly more robust with respect to the cumulative reward-based formulation of the hypothesis. This goes with the fact that the reward property neglects the frequent fluctuations in the given time horizon.

When focusing on the phenomenon of bistability, we can conclude that the most significant variance in the molecule population with respect to the two stable modes is observed in the range 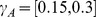 with 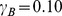. Here the distribution of the behaviour targeting the low and high mode is diversified nearly uniformly (especially for 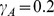). Note that in this case there is a significant amount of behaviour (around 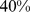) not converging to either of the two modes.

**Figure 8:**
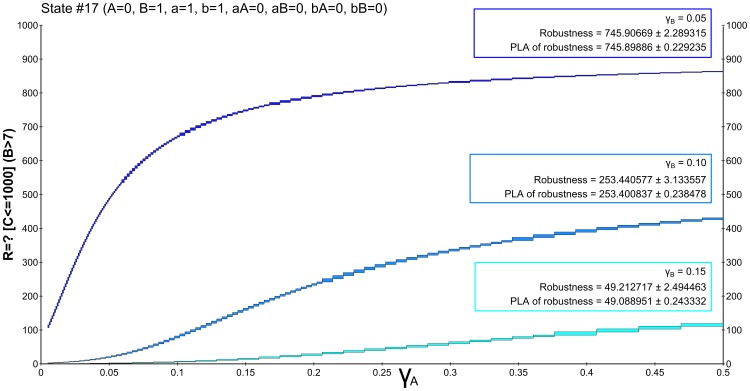
Results of robustness analysis for hypothesis 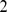. Hypothesis (2) requires stabilisation of 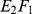 in the high concentration mode (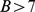). A CSL formula with cumulative reward operator is employed. Each of the curves represents the evaluation function over 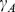 degradation obtained for a particular setting of 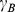. The horizontal axis shows the perturbation of *pRB* degradation rate and the vertical axis shows the probability of the hypothesis to be satisfied. In the upper left corner, robustness values are shown for each of the curves. The values are displayed with the absolute error quantifying the precision of the approximate method. For comparison, the values are computed also on piece-wise affine approximations of the evaluation function. It can be seen that the robustness values change rapidly with different settings of 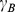. This observation goes with the fact that with faster degradation of 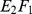 there is a lower probability that the positively self-regulated protein is locked in the stable mode of no production. In particular, the high stable mode is preferred for lower values of 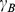. The increase of the value with increasing 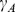 is due to the weakening effect of inhibition by *pRB*.

To encode the hypothesis 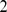 we employ the reward-based formulation: 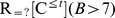. The time interval is set to be the same as in the previous case (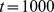). The resulting landscape visualisations for individual settings of 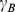 are depicted in Figure 8. It can be observed that the effect of 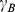 is now inverse, which goes with the fact that higher rate of 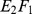 degradation causes the rapid dynamics of the protein and decreases the amenability of the cell to commit to *S*-phase (by making the hypothesis 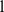 more robust than hypothesis 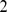).

**Figure 9:**
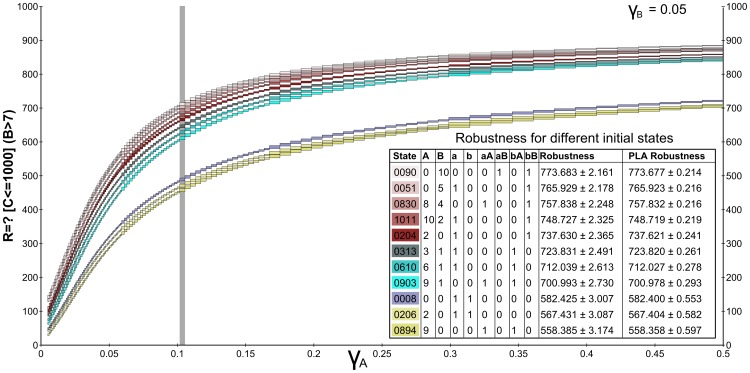
Landscape visualisation for hypothesis (2) and several selected initial states. The landscape visualisation of hypothesis (2) (stabilisation of 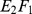 in the high concentration mode 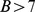) is shown for several selected initial states of the whole state space. A CSL formula with cumulative reward operator is employed. Each of the curves represents the evaluation function over 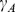 degradation obtained for a particular initial state and 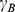 set to 0.05. The legend shows the amount of individual species in particular initial states and the robustness of the hypothesis is given together with the absolute error. The results obtained by piece-wise affine approximation are also shown. It can be seen that the hypothesis is only negligibly sensitive to initial conditions. Especially, only states with zero initial concentration of 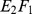 cause 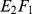 to attain low molecular numbers, thus lowering the robustness of the hypothesis. The grey vertical line shows the small perturbation in 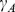 which is further explored in detail in Figure 10.

An interesting observation resulting from the analysis is that the selection of the initial state has only a negligible impact on the result. This is exploited in Figure 9 where we have selected 11 states uniformly distributed throughout the state space. Although low initial numbers of *B* slightly decrease robustness of hypothesis (2), the difference is not very big.

**Figure 10:**
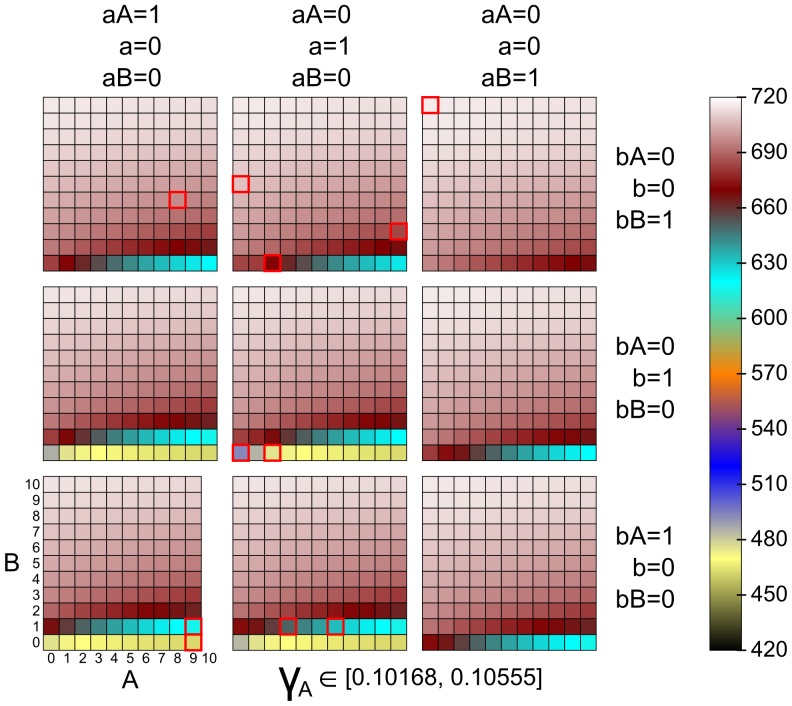
Analysis of hypothesis (2) for all initial states. Hypothesis (2) (stabilisation of 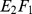 in the high concentration mode 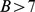) is computed and visualised for all initial states in the considered perturbation space 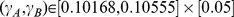. Because we assume at most a single molecule of DNA in the system, state variables denoting genes and gene-protein complexes have a binary domain. There are only two variables having a larger domain (0–10), in particular, these are the proteins *pRB* and 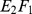. Therefore each of the (binary) combinations is visualised for the entire domain of *A* and *B* in a separate box. The colour intensity of each box in the grid shows the upper bound of the cumulative reward evaluated for the respective initial state. It can be seen that the hypothesis is mostly insensitive to the selection of initial states. Only the initial zero level of 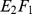 (*B, bB, aB*) causes a decrease of the resulting value. States selected in Figure 9 are highlighted in red.

More detailed insight can be inferred from Figure 10, where the evaluation of hypothesis (2) is exploited for a small perturbation of 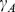 with respect to the entire initial state space. The considered perturbation is highlighted in Figure 9 by the grey vertical line. The colour intensity of the grid shows the upper bound of the cumulative reward evaluated for the respective initial state. It can be seen that the hypothesis is in most cases insensitive to the selection of initial states. Only the initial zero level of *B* causes a decrease of the resulting value. Moreover, this happens (naturally) just in two kinds of states: (*i*) no molecule of *B* is bound to any of the genes, i.e., the self-activation of *b* is inactive and the expression of *b* occurs in the spontaneous mode having a low rate 0.05; (*ii*) a molecule of *A* is bound to *b* thus imposing the inhibition on *b* and causing the same scenario.

### Robustness of Two-Component Signalling Systems Response

Signalling pathways make the main interface between cells and their environment. Their main role is to monitor biochemical conditions outside the cell and to transfer this information into the internal logical circuits (gene regulation) of the cell. Since signal processing is carried out by several dedicated protein complexes (signalling components), it is naturally amenable to intrinsic noise in these protein populations caused by stochasticity of transcription/translation processes. Robust input-output signal mapping is crucial for cell functionality. Many models and experimental studies have been conducted attempting to explain mechanisms of robust signal processing in procaryotic cells, e.g., [49], [50].

**Figure 11:**
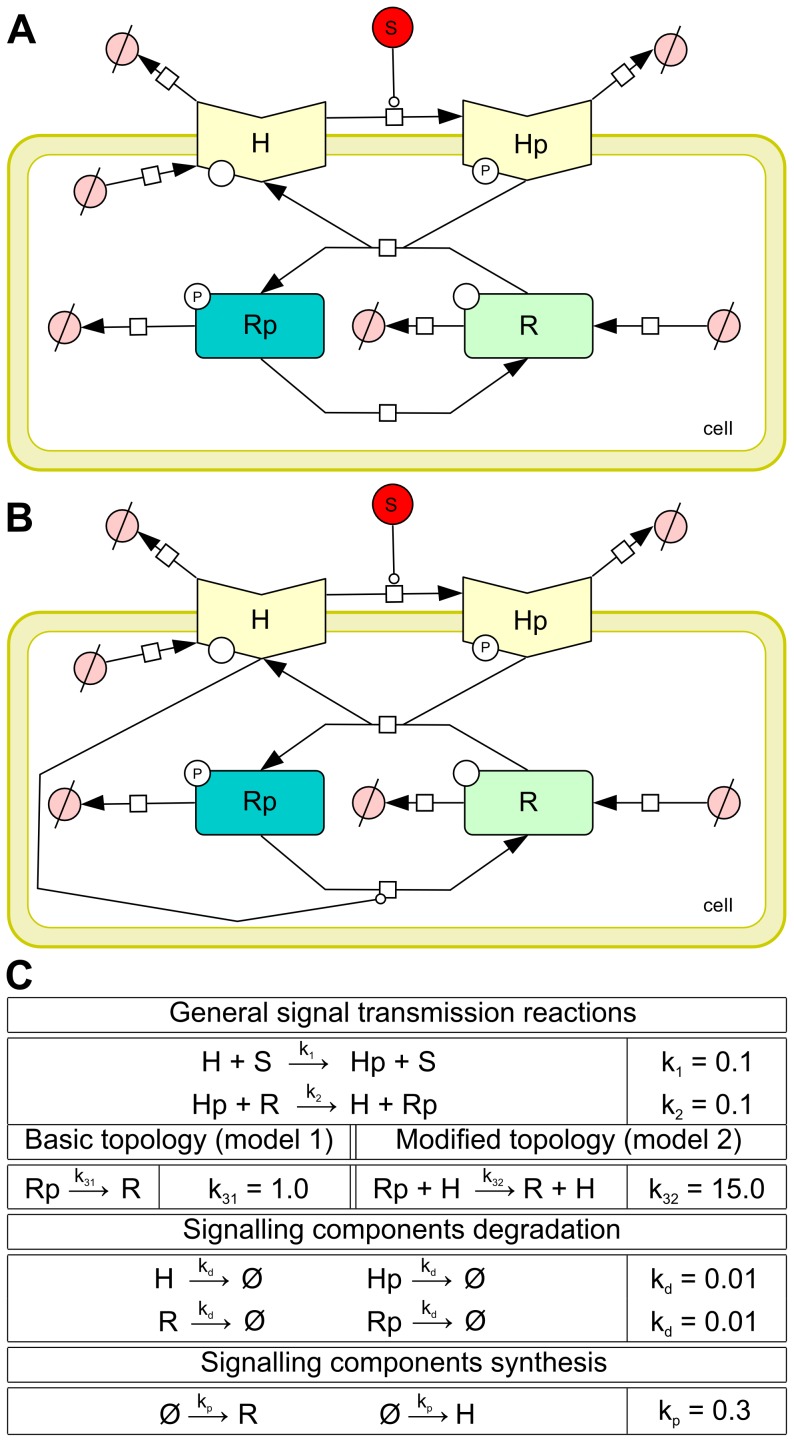
Model of a two-component signalling pathway. (A) Basic topology of the two-component signalling pathway. (B) Modified topology of the two-component signalling pathway, additionally, histidine kinase *H* catalyses dephosporylation of the response regulator *R*. (C) Reactions specifying the biochemical model of the two considered topologies of the two-component signalling pathway. Phosphorylation of the first component *H* catalysed by the input signal *S* and phosphorylation of the second component *R* are shared by both topologies, the only difference is in the second component’s dephophorylation. Additionally, we consider unregulated proteosynthesis/degradation reactions for both topology variants. Reaction topology in (A) and (B) was created using CellDesigner [54].

In order to construct robust signalling circuits in synthetically modified procaryotic cells, Steuer et al. [10] has suggested and analysed a modification of a well-studied two-component signalling pathway that is insensitive to signalling components concentration fluctuations. The study was conducted using a simplified model consisting of the two signalling components each considered in both phosphorylated and unphosphorylated forms. The first component, the histidine kinase *H*, is a membrane-bound receptor phosphorylated by an external signalling ligand *S*. In its phosphorylated form *Hp*, the histidine kinase transfers the phospho-group onto the second component – the response regulator *R*. That way it activates the response regulator by transforming it into the phosphorylated form *Rp*, which is diffusible and functions as the internal signal for the cell. The basic topology of the pathway is depicted in Figure 11A. The modification suggested by Steuer et al. is depicted in Figure 11B. The difference is in the addition of catalytic activation of *Rp* dephosphorylation by the unphosphorylated histidine kinase *H*. In [10] it has been rigorously proven that under the deterministic setting this modification leads to globally robust steady-state response of the signalling pathway, which is not achievable with the basic topology.

We reformulate the model in the stochastic setting and employ our method to provide detailed analysis of the input-output signal response under fluctuations in population of both signalling components. In contrast to [10], where the average steady-state population is analysed with respect to fluctuations in signalling components, our analysis refines the steady population in terms of distributions. That way we obtain for a stable input signal a detailed view of distribution of the output response. In particular, instead of studying the effect of perturbations on the average population, we see how perturbations affect the distribution, i.e., the variance (fluctuation) in the output response. That way the stochastic framework gives a more detailed insight into the input-output signal response mechanism.

The biochemical model of both topology variants is given in Figure 11C. The input signal *S* is considered to be fixed and therefore it makes a constant parameter of the model. The signalling components in both phosphorylated and unphosphorylated forms make the model variables *H, Hp, R*, and *Rp*.

Depending on which topology is chosen, the original deterministic model [10] exhibits different relationships between the steady-state concentrations of the input signal *S* and the output signal *Rp*:
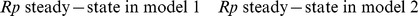





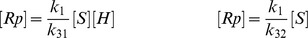



In particular, it can be seen that the steady-state concentration of the output signal [*Rp*] in model 1 is affected not only by the input signal *S* but also by the number of unphosphorylated receptors *H*, which can be interpreted in such a way that the concentration of the signalling components should be kept stable in order to obtain a robust output. This is, however, not an issue in model 2 where *Rp* depends only on *S*. Since the steady-state analysis has been carried out under the deterministic setting additionally imposing assumptions of conserved total amounts of *H+Hp* and *R+Rp*, it is appropriate only for high molecular populations.

The question we want to answer is “Is there a difference in the way the two models handle noise (fluctuations) for low molecular numbers of signalling components?” In such conditions, populations of *H+Hp* and *R+Rp* cannot be considered conserved, since the proteins are subject to degradation and production. Production of proteins from genes, as well as degradation, is inherently noisy as demonstrated in the previous case study. Different levels of noise can be affected by, e.g., regulatory feedback loops or varying numbers of gene copies. Even for a noiseless output signal *S* these internal fluctuations of protein concentrations transfer noise to *Rp*. We formalise our question in terms of the CSL property 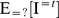, which asks for the value of a post-processing function in a future time *t*, where the post-processing function is defined as the *mean quadratic deviation* of the distribution of *Rp*.

For the model to have low numbers of molecules exhibiting stochastic fluctuations and to enable responses to varying levels of *S*, we have chosen 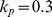 *molecules
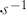
* and 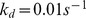, which leads to an average total population of 30 molecules for both 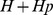 and 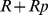. To make the analysis straightforward we assume the same speed of degradation of phosphorylated and unphosphorylated variants of each protein.

**Figure 12:**
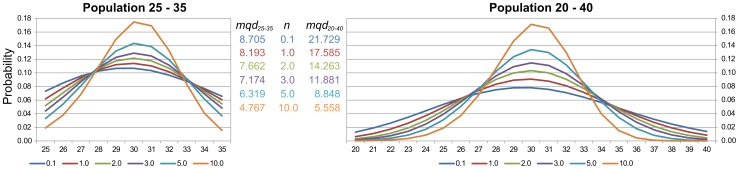
Influence of state space truncation to mean quadratic deviation of a distribution. A simple birth death model is considered to show the influence of different settings of the state space truncation on the measured noise evaluated in the form of a *mean quadratic deviation* (*mqd*) of the state space distribution. The model has a single species *X* and two reactions 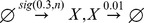 which stabilise the population around an average of 30. For different values of the sigmoid coefficient *n* we can see different *mqd* values, the larger the *n* the smaller the noise. If *X* is restricted to 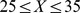 the overall noise is smaller since the probability mass cannot spread to states placed further from the mean. In a less restricted version with populations between 20 and 40 the noise is about 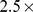 larger. If the sigmoid regulation is weak and the regulation is strong then the difference in the amount of noise is less than 20%.

To reduce the size of the state space we have truncated total populations to 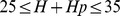 and 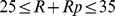, which leads to 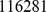 states in total. The initial state is considered with populations 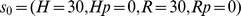. The state space reduction has a significant impact on the measured absolute values of noise but conserves general trends as is shown in Figure 12.

In order to control fluctuations in protein production we extend our model with two populations of genes, one for *H* and one for *R*, respectively, and for each of the genes we introduce an autoregulatory negative feedback loop via binding the proteins to their corresponding genes. That way we restrict the protein production. By modifying the number of gene copies in the cell and the rate of protein-gene binding, we are able to regulate the overall noise in the transcription. This approach, however, significantly increases the state space size because it introduces new variables representing genes and protein-gene complexes. To make the analysis feasible, we abstract from details of the underlying autoregulatory mechanism and model it using a sigmoid production function, which mimics the desired behaviour accordingly. Using numerical analysis, we have verified that such an approximation can be employed in the stochastic framework. The function is defined in the following way:
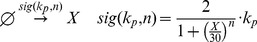
where 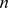 is the so-called Hill coefficient controlling the steepness of the sigmoid (caused by cooperativity of transcription factors in protein-gene interactions) and 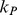 is the maximal production rate. We use this approach for modelling the production of both species *H* and *R* by sigmoid coefficients denoted 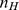 and 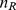, respectively. The sigmoid function regulates the population by enabling production when it is below average and repressing it when the population is above the average. Larger *n* is leads to steeper sigmoid functions, which leads to stronger regulation and lower noise. The case *n = 0* corresponds to an unregulated model and when increased to *n = 20* it corresponds to over 10 copies of each gene in the fully modelled feedback loop mechanism. The effect of different levels of sigmoid regulation to noise can be seen in a simplified birth death model in Figure 12.

To see the long-term effects of intrinsic noise we decided to examine the system in the situation when the output response is stabilised. Since the min-max approximation method cannot be employed with steady-state computation, transient analysis in a suitable time horizon has been performed instead. To estimate the closest time *t* when the system’s behaviour can be observed as stable, we have computed values of output response noise for the unregulated variant of the model (*n = 0*) using standard numerical steady state numerical analysis (we employed the tool PRISM [44]) and compare it to probability distributions obtained by transient analysis in 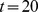, 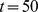 and 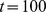 seconds. Consequently, we have compared the probability distribution in the steady state with the probability distribution in 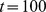 seconds. The results clearly show that the difference in distributions is negligible and the transient distribution can be considered stable after 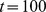.

To further speed up the computation, we have precomputed the distribution of *H* and *R* in the time horizon 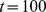 without enabling phosphorylation reactions. This has led to a significant reduction to 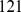 states. Starting with the achieved probability distribution, we have subsequently computed the transient analysis with enabled phosphorylation reactions in the next 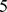 seconds. The rationale behind is that the protein production and degradation are two orders of magnitude slower than phosphorylation. Therefore, the total populations of *H* and *R* dictate the time at which the system is nearly stable and thus the next 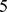 seconds are sufficient for the fast-scale phosphorylation to stabilise the fractions 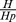 and 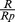.

To compute the noise (variance) in *Rp* we employ the *mean quadratic deviation* post-processing function for state space distributions. Our goal is to compare the levels of *Rp* noise in both models for different levels of the output signal *S* and for different values of intrinsic noise appearing in protein production (controlled by sigmoid coefficients 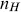 and 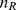). After computing lower and upper bounds of the state space distributions, we have computed the lower and upper bounds of the post-processing function using the algorithm informally introduced in Section Parameterised Uniformisation. Consequently, we obtain robustness values for the output response 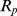 over the respective perturbation subspaces in the form *average* 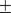 *error*. Finally, we define the perturbation space of the interest. In particular, for the signal we choose the value interval 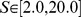 and for sigmoid coefficients 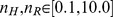.

**Figure 13:**
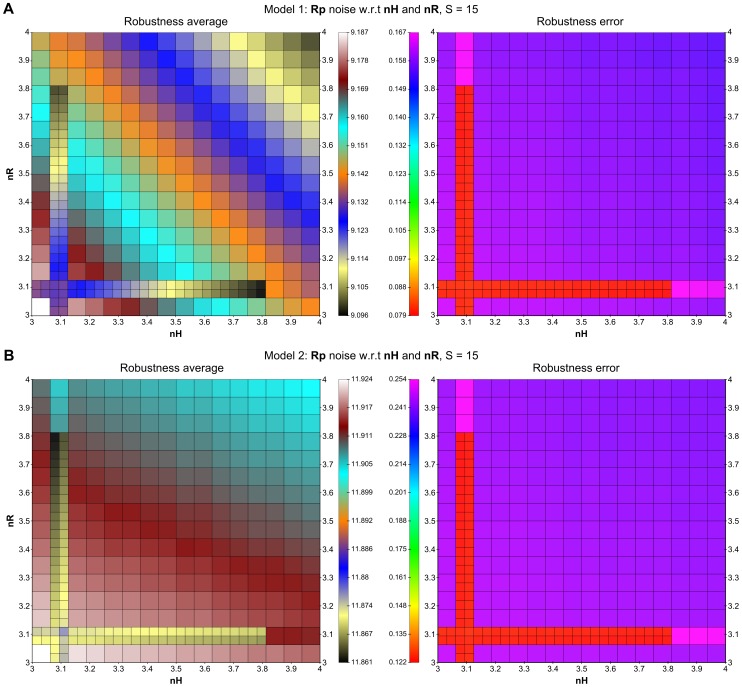
Influence of genetic regulation on noise in model 1 and 2. In the upper part, the *Rp* noise in model 1 is computed over perturbations of both sigmoid production constants 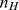 and 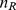 in 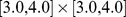. The upper and lower bounds on noise (mean quadratic deviation of the resulting probability distribution projected onto populations of *Rp*) are recomputed into the form *average* 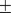 *error*, the average values are shown on the left and errors are shown on the right. The densely subdivided subspaces around the value 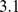 are due to conservative over/under approximations in the computation of the probability distribution in states where inflow and outflow of the probability mass is not strictly a monotonous function over the given perturbation interval, thus the error is locally increased and the subspaces must be further divided to obtain the required precision. In the lower part, the *Rp* noise in model 2 is computed. By comparing both results we can make two observations: a) Model 1 has an overall lower noise and also the computation error, given the same level of refinement. b) In model 1 the results are symmetrical with respect to perturbations in 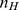 and 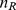, with 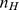 having a slightly larger influence, but in model 2 the results are not symmetrical and 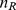 has a larger influence. However, we considered the difference negligible and combined both parameters into a single sigmoid production constant *n*.

Since the full computation over the 3-dimensional perturbation space has turned out to be intractable, we have to find a way to reduce its dimension. To this end, we focus on a subspace 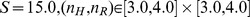 where both models have symmetric sensitivity to both sigmoid production coefficients 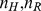. This symmetry allows us to merge 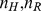 into a single coefficient *n*. Results of this experiment are visualised in Figure 13, where it can be seen that in Model 1 the influence of 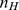 and 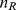 is almost perfectly symmetrical with 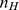 being slightly more influential. In Model 2 the influence is evidently stronger in 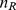 but the response seems to be symmetrical enough to justify the sigmoid coefficients merging. An interesting property of the parameterised uniformization and the perturbation space decomposition algorithm can be seen in Figure 13, where the decomposition of the perturbation spaces around both sigmoid coefficients set to 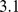 is very dense. This is due to the non-linearity of the sigmoid production functions, which leads to the non-monotonicity of probability inflow/outflow differences in states during parameterised uniformisation (see Section Methods). In order to preserve the conservativeness of estimates we have to locally over/under approximate these inflow/outflow rates thus gaining an increase of error. To obtain the desired level of accuracy, we dynamically refine all those subspaces where this has occurred.

**Figure 14:**
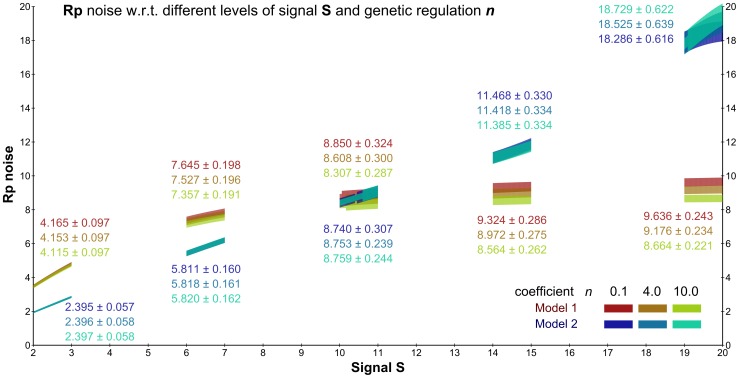
Comparison of models by *Rp* noise robustness. Robustness *Rp* noise in both models has been computed with respect to perturbations of signal *S* over five selected intervals of the input signal 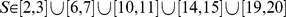 and for three distinct levels of the intrinsic noise in signalling component dynamics represented by sigmoid coefficient 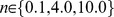. Perturbations were not computed over the whole interval 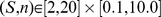 due to high computational demands. From the computed values of individual refined subspaces as well as the aggregated robustness values for each input signal interval we can see that for lower values of signal *S* (up-to 10), Model 2 embodies lower output response noise than Model 1 (spontaneous dephosphorylation). While the output response noise in Model 1 tends to converge to values between 8 and 10, Model 2 exhibits a permanent (almost linear) increase in the output response noise over most of the studied portion of the perturbation space. A super-linear increase of the noise is observed for strong input signals. Another interesting aspect is that, with increasing levels of gene regulation given by sigmoid coefficient *n*, the overall noise in *Rp* decreases over the whole interval of signal values for Model 1 and most of the interval for Model 2. However, there is an anomaly in Model 2 in the high signal region [19.0, 20.0], where with decreasing noise in *R* and *H* (see Figure 15) the noise in *Rp* increases.

**Figure 15:**
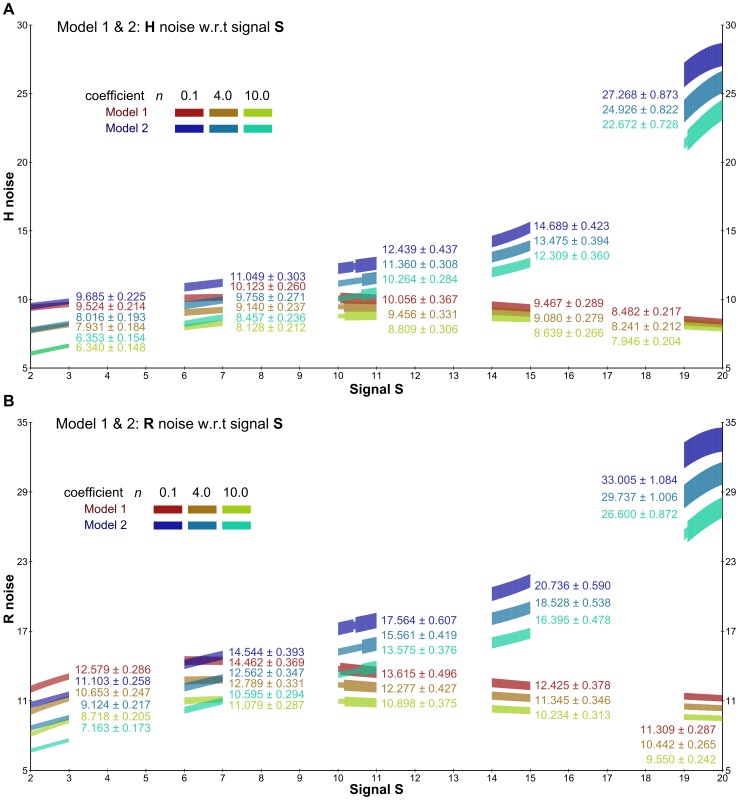
Noise in populations or *H* and *R* in both models. Noise in *H* (A) and *R* (B) in both models has been computed with respect to perturbations of signal *S* over five selected intervals 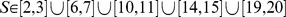 and for three distinct levels of inherent production noise represented by sigmoid coefficient 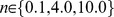. We can see that in all cases, with increasing regulation by *n*, the intrinsic noise in the dynamics of each of the signalling components decreases.

**Figure 16:**
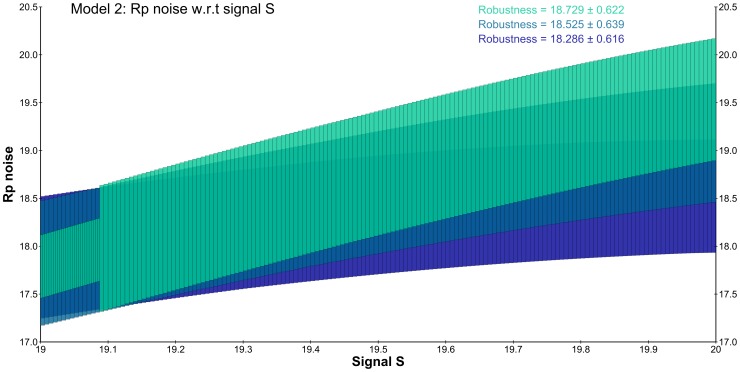
High signal region in Model 2. A magnification of the high signal region in Model 2, where increasing levels of regulation by the sigmoid coefficient *n* leads to a paradoxical increase of output response noise instead of a decrease. Even though the inaccuracy is large we consider the trend to be strong and thus real.

Finally, we inspect selected subintervals of the perturbation space given by five exclusive intervals of the input signal value domain, 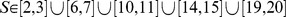, and three distinct levels of production noise represented by sigmoid coefficient 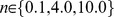. The results of this main experiment can be seen in Figure 14 and Figure 15. The trends that can be seen in Figure 14 are that for lower signals up to *S = 10*. Model 2 has encountered lower noise in *Rp* than Model 1 but in the higher signal region it is outperformed by Model 1, which quickly converges to values between 8 and 10. However, *Rp* noise produced in Model 2 linearly increases with increasing value of the input signal *S*. For most of the inspected subspaces a stronger regulation of *H* and *R* production by the sigmoid coefficient *n* leads to a reduction of *Rp* noise. An exception to this observation can be seen in Model 2 at the signal interval 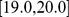 where this trend is inverted. To show that this is an emergent behaviour arising from non-trivial interaction between phosphorylation and dephosphorylation reactions not present in the production and degradation of components *H* and *R*, their respective influences are displayed in Figure 15. There we can see that in Model 1 both *H* and *R* follow an initial increase of noise with increasing *S* but then the noise stabilises. This leads us to a hypothesis that the regulation of noise in signalling components dynamics loses its influence as signal *S* increases. This is however due to the fact that more *S* leads to faster phosphorylation of *H*, which effectively reduces the population of *H* thus also reducing its absolute noise. In the case of Model 2 the situation is different since we can observe a permanent increase of noise in both *H* and *R* populations. The inversion of noise with increased regulation seen in Figure 14 and magnified in Figure 16 has not yet been explained satisfactorily.

## Discussion

In this paper we proposed a novel framework for robustness analysis of stochastic biochemical systems. It allows us to quantify and analyse how the validity of a hypothesis formulated as a temporal property depends on the perturbations of stochastic kinetic parameters and initial populations. The framework extends the quantitative model checking techniques and numerical methods for CTMCs and adapts them to the needs of stochastic modelling in biology. Therefore, in contrast to statistical techniques such as Monte Carlo simulation, parameter sampling and adaptive grid refinement (recently used in [28]), our framework is customisable with respect to the required precision of computation. This is obtained by providing the lower and upper bounds of the results that allow us to rigorously focus on a considered perturbation space of interest and to provide detailed analysis of the evaluation function.

It is worth noting that the evaluation function can be discontinuous or may change its value rapidly on a very small perturbation interval in situations when the given CSL formula contains nested probability operators. In particular, this leads inevitably to the formulation of a hypothesis requiring a detailed temporal program [51] of the biological system (e.g., temporal ordering of events). This makes another reason why we need to guarantee the approximated shape of the evaluation function.

Case studies have demonstrated that the framework can be successfully applied to the robustness analysis of nontrivial biochemical systems. They have shown how to use CSL to specify properties targeting transient behaviour under fluctuations. From the first case study we can conclude that the reward-based formulation of stability properties is more appropriate for distinguishing the individual parameter settings under therequested range of uncertainty than the formulation using the globally operator. The inspected biological hypothesis in the second case study cannot be directly formulated using CSL with rewards. Therefore, we have employed post-processing functions to express and study the mean quadratic deviation of the molecule population distribution of the signal response regulator protein.

The time complexity of our framework in practice depends mainly on the size of the state space, the number of reaction steps that have to be considered, and the number of perturbation sets that have to be analysed to provide the desired precision. The size of the state space is given by the number of species and their populations. The framework is suitable for low populations and is relevant especially in the case of gene regulation. In the first case study we have considered only a single molecule of DNA and thus the state space of resulting CTMC was manageable. In the second case study we have abstracted the feedback loop mechanism using a sigmoid production function to reduce the state space and to make the analysis feasible. If such an abstraction cannot be used, our framework can be effectively combined with general state space reduction methods for CTMCs, e.g., finite projection techniques [11], [12], dynamic state space truncation [13], and aggregation methods [52]. The number of reaction steps can be reduced using separation of fast and slow reactions as demonstrated in the second case study or using adaptive uniformisation [13], [53].

In the first case study several hundreds of perturbation subsets had to be analysed and the overall robustness analysis took a few hours. However, in the second case study several thousands of perturbation subsets where required to achieve reasonable precision. In order to speedup the computation we analysed the subsets in parallel using a high-performance multi-core workstation were the analysis took several hours. To further improve the accuracy of the robustness analysis without decreasing the performance, we have employed a piece-wise linear approximation. It allows us to obtain more precise results without increasing the number of perturbation sets, yet it does not guarantee conservative error bounds.

The presented method as employed in the first case study gives us a tool for exact analysis of bistability from the global point of view (with respect to all initial conditions, the considered time bound, and the given range of parameters). It can be considered as an analogy to bifurcation analysis known from the ODE world. When comparing our approach with the bifurcation analysis performed in [9], our approach provides a detailed mesoscopic insight into the analysed phenomenon. Instead of identifying just the points where the population diverges, we obtain the precise knowledge of how the population is distributed around the two stable states. Especially, the method shows that reachability of the cancer-inducing high stable mode of the retinoblastoma-binding transcription factor is almost always possible despite the initial state of the regulatory system. An exhaustive analysis is performed with uncertainty in the degradation parameters of the two most important cell-cycle regulating proteins. However, if the degradation of the tumour suppressor protein is sufficiently high, there is always a possibility allowing the population to switch into the safe low stable mode. Moreover, the robustness of having the possibility to avoid the cell malfunction is positively affected by increasing the retinoblastoma-binding transcription factor degradation. In contrast to [9], the switching mechanism is described at the single cell level, which allows to quantify the portion of population amenable to malfunction and thus can provide a preliminary guide to further analysis, targeting elimination of the undesired behaviour.

The second case study shows new insights into the phenomenon of noise in two-component signalling pathways appearing in procaryotic organisms. The previous study [10] conducted in the framework of deterministic models targeted global robustness of steady state concentrations of output signalling components by means of analytically finding the invariant perturbation space. The result has shown that a synthetic pathway topology, including additional catalysis of signal response regulator by histidine kinase, leads to globally robust input-output signal mapping with respect to fluctuations in signalling components concentration. On the other hand, the basic topology without histidine-modulated dephosphorylation does not fulfil global robustness. Since signalling pathways are understood to be amenable to intrinsic noise due to relatively low molecule populations of signalling proteins (typically hundreds of molecules), the respective stochasticity might affect the input-output signal response. To this end, we have reformulated the model in the stochastic framework and instead of studying the effect of perturbations on the average population, we study in detail how perturbations affect the distribution, i.e., the variance (fluctuation) in the output response. Our study shows that both pathway topologies result in fluctuations of the output response, but robustness of input-output mapping varies in both models with increasing the level of the (constant) input signal. For low input signals the synthetic topology gives response with smaller variance in the output, whereas for high input signals the output variance rapidly increases. Therefore the basic topology seems to be more suitable for processing strong signals while the synthetic topology is more appropriate for low level signals. Our study has also shown that both topologies are quite robust with respect to scaling the noise in signalling components dynamics.

Although the results presented in Figures 14 and 15 do not cover full ranges of the signal and the gene regulation, they allow us to capture the important trends in the behaviour of both topologies. The computation over the full ranges would significantly increase the computational demands. However, our aim was not to provide the result for the full range but rather to rigorously analyse the robustness for different levels of the signal and the gene regulation. Currently, we focus on an extension of our framework employing statistical techniques. This extension has the potential to efficiently scan multidimensional parameter spaces and to identify interesting subspaces that can be analysed in detail using the framework.

## Supporting Information

Text S1 **Methodology description.** A detailed description of formal methods employed in the framework for robustness analysis of stochastic biochemical systems.
(PDF)
