# Supplementary material for: Robustness Analysis of Stochastic Biochemical Systems
Source: PLoS One. 2014 Apr 21;9(4):e94553. doi: 10.1371/journal.pone.0094553 (PMC3994026; doi:10.1371/journal.pone.0094553)
Supplement: Text S1 — Methodology description. A detailed description of formal methods employed in the framework for robustness analysis of stochastic biochemical systems. (PDF) [file pone.0094553.s001.pdf]

Text S1 - Methodology Description

Milan Češka, David Šafránek, Sven Dražan and Luboš Brim

I Continuous Stochastic Logic

Let $\mathcal{C} = (\mathbb{S}, s_0, \mathbf{R}, L)$ be a labelled CTMC such that L is a labelling function which assigns to each state $s \in \mathbb{S}$ the set *L(s)* of atomic propositions that are valid in state s. We consider the specification of the inspected property using the bounded time fragment of CSL with rewards and post-processing functions. The syntax of this logic is defined in the following way. A state formula Φ is given as

$$\Phi ::= \mathsf{true} \mid a \mid \neg\Phi \mid \Phi \wedge \Phi \mid \mathsf{P}_{\sim p}[\phi] \mid \mathsf{R}_{\sim r}[\mathsf{C}^{\leq t}] \mid \mathsf{R}_{\sim r}[\mathsf{I}^{=t}] \mid \mathsf{E}_{\sim r}[\mathsf{I}^{=t}]$$

where ϕ is a path formula given as $\phi ::= \mathsf{X}\ \Phi \mid \Phi\ \mathsf{U}^I\ \Phi$, a is an atomic proposition, $\sim \in \{<, \leq, \geq, >\}$, $p \in [0,1]$ is a probability, $r \in \mathbb{R}_{\geq 0}$ is an expected reward and $I = [a, b]$ is a bounded time interval such that $a, b \in \mathbb{R}_{\geq 0} \wedge a \leq b$. Path operators G (always) and F (eventually) are derived in the standard way using the operator U. In order to specify properties containing rewards ($\mathsf{R}_{\sim r}[\mathsf{C}^{\leq t}]$ is the *cumulative reward* acquired up to time t, $\mathsf{R}_{\sim r}[\mathsf{I}^{=t}]$ is the *instantaneous reward* in time t) the CTMC $\mathcal{C}$ is enhanced with reward (cost) structures. Two types of reward structures are used. *A state reward* $\rho(s)$ defines the rate with which a reward is acquired in state $s \in \mathbb{S}$. A reward of $t \cdot \rho(s)$ is acquired if a CTMC remains in state s for t time units. *A transition reward* $\iota(s_i, s_j)$ defines the reward acquired each time the transition (s_i, s_j) occurs.

Since the reward function has to be defined before the actual analysis of the CTMC, the rewards for particular states have to be known prior to the specification of the property. This fact limits the class of properties that can be expressed using such structures. For example, noise expressed by a *mean quadratic deviation* (*mqd*) of the population probability distribution of a species at a given time cannot be specified using CSL with rewards. To compute the *mqd* we need to know the mean of the distribution to be able to obtain the corresponding coefficients and encode them into state rewards.

To overcome this problem we introduce the abstract state operator $\mathsf{E}_{\sim r}[\mathsf{I}^{=t}]$ which *evaluates* the state distribution $\pi^{\mathcal{C},s_0,t}$ at the given time instant t by a user provided real-valued *post-processing* function $Post(\pi^{\mathcal{C},s_0,t})$ and compares it to $\sim r$.

The formal semantics of the bounded fragment of CSL with rewards and post-processing functions is defined similarly as the semantics of full CSL and thus we refer the readers to original papers. The key part of the semantics is given by the definition of the satisfaction relation $\models$. It specifies when a state s satisfies the state formula Φ (denoted as $s \models \Phi$) and when a path ω satisfies the path formula ϕ (denoted as $\omega \models \phi$). The informal definition of $\models$ is as follows:

- $s \models \mathsf{E}_{\sim r}[\mathsf{I}^{=t}]$ iff $Post(\pi^{\mathcal{C},s,t})$ satisfies $\sim r$.
- $s \models \mathsf{P}_{\sim p}[\phi]$ iff the probability of all paths $\omega \in Path^{\mathcal{C}}(s)$ that satisfy the path formula ϕ (denoted as $Prob^{\mathcal{C}}(s, \phi)$) satisfies $\sim p$, where
  - ω satisfies $\mathsf{X}\ \Phi$ iff the second state on ω satisfies Φ
  - ω satisfies $\Phi\ \mathsf{U}^I\ \Psi$ iff there exists a time instant $t \in I$ such that the state on ω occupied at t satisfies Ψ and all states on ω occupied before $t' \in [0, t)$ satisfy Φ

- $s \models \mathsf{R}_{\sim r}[\mathsf{C}^{\leq t}]$ iff the sum of expected rewards over $Path^{\mathcal{C}}(s)$ *cummulated* until t time units (denoted as $Exp^{\mathcal{C}}(s, \mathsf{X}_{\mathsf{C}^{\leq t}})$) satisfies $\sim r$

- $s \models \mathsf{R}_{\sim r}[\mathsf{I}^{=t}]$ iff the sum of expected rewards over all paths $\omega \in Path^{\mathcal{C}}(s)$ at time t (denoted as $Exp^{\mathcal{C}}(s, \mathsf{X}_{\mathsf{I}^{=t}})$) satisfies $\sim r$.

A set $Sat_{\mathcal{C}}(\Phi) = \{s \in \mathbb{S} \mid s \models \Phi\}$ denotes the set of states that satisfy Φ.

Note that the syntax and semantics can be easily extended with "quantitative" formulae in the form $\Phi ::= \mathsf{P}_{=?}[\phi] \mid \mathsf{R}_{=?}[\mathsf{C}^{\leq t}] \mid \mathsf{R}_{=?}[\mathsf{I}^{=t}] \mid \mathsf{E}_{=?}[\mathsf{I}^{=t}]$, i.e., the topmost operator of the formula Φ returns a quantitative result, as used, e.g., in PRISM [1]. In this case the result of a decision procedure is not in the form of a boolean yes/no answer but is the actual numerical value of the probability $Prob^{\mathcal{C}}(s, \phi)$, the expected reward $Exp^{\mathcal{C}}(s, \mathsf{X})$ for $\mathsf{X} \in \{\mathsf{X}_{\mathsf{I}^{=t}}, \mathsf{X}_{\mathsf{C}^{\leq t}}\}$ or the value of $Post^{\mathcal{C}}(s, t)$. The computation of a numerical value is of the same complexity class as the computation of a result to be compared leading to a boolean answer, although in some cases the comparison may be carried out on less precise or preliminary results.

II Transient Analysis

The aim of transient analysis is to compute a transient probability distribution. Given an initial distribution $\pi^{\mathcal{C}, s_0, 0}$ (i.e., $\pi^{\mathcal{C}, s_0, 0}(s) = 1$ if $s_0 = s$, and 0, otherwise) at time 0 of a CTMC $\mathcal{C} = (\mathbb{S}, s_0, \mathbf{R})$ what will the transient state distribution $\pi^{\mathcal{C}, s_0, t}$ look like in some future yet finite time $t \in \mathbb{R}_{\geq 0}$.

Transient analysis of a CTMC may be efficiently carried out by a standard technique called *uniformisation* [2]. The transient probability in time t is obtained as a sum of expressions giving the state distributions after i discrete reaction steps of the respective *uniformised* discrete time Markov chain (DTMC) weighted by the ith probability of the Poisson process. It is the probability of i such steps occurring in time t, assuming the delays between steps of the CTMC $\mathcal{C}$ are exponentially distributed with *rate* q. Formally, for the rate q satisfying $q \geq max\{E^{\mathcal{C}}(s) \mid s \in \mathbb{S}\}$ (E is the exit rate of state s) the uniformised DTMC $\mathsf{unif}(\mathcal{C})$ is defined as $\mathsf{unif}(\mathcal{C}) = (\mathbb{S}, s_0, \mathbf{Q}^{\mathsf{unif}(\mathcal{C})})$ where

$$\mathbf{Q}^{\mathsf{unif}(\mathcal{C})}(s, s') = \begin{cases} \frac{\mathbf{R}(s,s')}{q} & \text{if } s \neq s' \\ 1 - \sum_{s'' \neq s} \frac{\mathbf{R}(s,s'')}{q} & \text{otherwise,} \end{cases}$$

and the ith Poisson probability in time t is given as $\gamma_{i,q \cdot t} = e^{-q \cdot t} \cdot \frac{(q \cdot t)^i}{i!}$. The transient probability can be computed as follows:

$$\pi^{\mathcal{C}, s_0, t} = \sum_{i=0}^{\infty} \gamma_{i,q\cdot t} \cdot \pi^{\mathcal{C}, s_0, 0} \cdot (\mathbf{Q}^{\mathsf{unif}(\mathcal{C})})^i \approx \sum_{i=L_\epsilon}^{R_\epsilon} \gamma_{i,q\cdot t} \cdot \pi^{\mathcal{C}, s_0, 0} \cdot (\mathbf{Q}^{\mathsf{unif}(\mathcal{C})})^i.$$

Although the sum is in general infinite, for a given precision ϵ the lower and upper bounds L_ϵ, R_ϵ can be estimated by using techniques such as that of Fox and Glynn [3] which also allow for efficient solutions of the Poisson process. In order to make the computation of uniformisation feasible the matrix-matrix multiplication is reduced to a vector-matrix multiplication, i.e.,

$$\pi^{\mathcal{C}, s_0, 0} \cdot (\mathbf{Q}^{\mathsf{unif}(\mathcal{C})})^i = (\pi^{\mathcal{C}, s_0, 0} \cdot (\mathbf{Q}^{\mathsf{unif}(\mathcal{C})})^{i-1}) \cdot \mathbf{Q}^{\mathsf{unif}(\mathcal{C})}.$$

Standard uniformisation can be intractable when the system under study is too complex, i.e., contains more than an order of 10^7 states and the upper estimate R_ϵ, denoting the number of vector-matrix multiplications as iterations, is high (more than an order of 10^6). Therefore, many approximation techniques have been studied in order to reduce the state space and to lower the number of iterations R_ϵ. State space reductions are based on the observation that in many cases (especially in biochemical systems) a

significant amount of the probability mass in a given time is localised in a manageable set of states. Thus neglecting states with insignificant probability can dramatically reduce the state space while the resulting approximation of the transient probability is still sufficient. Methods allowing efficient state-space reduction are based on finite projection techniques [4, 5], dynamic state space truncation [6] and aggregation methods [7].

Since the number of iterations R_ϵ inherently depends on the uniformisation rate q that has to be greater than the maximal exit rate of all the states of the system, a variant of standard uniformisation, the so-called *adaptive* uniformisation [8], has been proposed. It uses a uniformisation rate that adapts depending on the set of states the system can occupy at a given time, i.e., after a particular number of reactions. In many cases, a significantly smaller rate q can be used and thus the number of iterations R_ϵ can be significantly reduced during some parts of the computation. Moreover, adaptive uniformisation can be successfully combined with reduction techniques mentioned above [6]. The downside of adaptive uniformisation is that the Poisson process has to be replaced with a general *birth* process which is more expensive to solve. More details can be found, e.g., in [8].

III Global CSL Model-Checking

Let us first define an auxiliary function $Eval(\mathcal{C}, \Phi)$, that returns the numerical value representing the quantitative model-checking result for a given CTMC $\mathcal{C} = \{\mathbb{S}, s_0, \mathbf{R}\}$ and an inspected formula Φ, in the following way:

$$Eval(\mathcal{C}, \Phi) = \begin{cases} Prob^{\mathcal{C}}(s_0, \phi) & \text{if } \Phi \equiv \mathsf{P}_\star[\phi] \\ Exp^{\mathcal{C}}(s_0, \mathsf{X}_{\mathsf{C}^{\leq t}}) & \text{if } \Phi \equiv \mathsf{R}_\star[\mathsf{C}^{\leq t}] \\ Exp^{\mathcal{C}}(s_0, \mathsf{X}_{\mathsf{I}^{=t}}) & \text{if } \Phi \equiv \mathsf{R}_\star[\mathsf{I}^{=t}] \\ Post(\pi^{\mathcal{C}, s_0, t}) & \text{if } \Phi \equiv \mathsf{E}_\star[\mathsf{I}^{=t}] \end{cases} \tag{1}$$

where $\star \in \{=?, \sim r\}$.

The goal of the local model checking technique is to compute the value $Eval(\mathcal{C}, \Phi)$. On the other hand, for the global model checking technique it is to compute the values of $Eval(\mathcal{C}, \Phi)$ for all $\mathcal{C} = \{\mathbb{S}, s, \mathbf{R}\}$ where $s \in \mathbb{S}$. The crucial advantage of the global approach is the fact that it has the same computational complexity as the local approach. Therefore, the global model checking technique is much more suitable for robustness analysis over perturbations of initial conditions that are encoded as the initial state of the corresponding CTMC.

Global model checking returns the vector of size $|\mathbb{S}|$ such that the ith position contains the model checking result provided that s_i is the initial state. Let $\mathcal{C} = (\mathbb{S}, \mathbf{R}, L)$ be a labelled CTMC where the initial state is not specified. The crucial part of this method is to compute the vector of probabilities $\overline{Prob}^{\mathcal{C},\phi}$ for any path formula ϕ and the vector of expected rewards $\overline{Exp}^{\mathcal{C},\mathsf{X}}$ for $\mathsf{X} \in \{\mathsf{X}_{\mathsf{I}^{=t}}, \mathsf{X}_{\mathsf{C}^{\leq t}}\}$ such that for all $s \in \mathbb{S}$ the following holds:

$$\overline{Prob}^{\mathcal{C},\phi}(s) = Prob^{\mathcal{C}}(s, \phi) \wedge \overline{Exp}^{\mathcal{C},\mathsf{X}}(s) = Exp^{\mathcal{C}}(s, \mathsf{X})$$

In local model checking the computation of $Prob^{\mathcal{C}}(s, \phi)$ and $Exp^{\mathcal{C}}(s, \mathsf{X})$ is reduced to the computation of the transient probability distribution $\pi^{\mathcal{C},s,t}$, as described, e.g., in [2,9]. Thus, for different initial states s we have to compute the corresponding transient probability distributions separately. The key idea of the global model checking method is to use *backward transient analysis.* The result of backward transient analysis is the vector $\tau^{\mathcal{C},\mathbb{A},t}$ such that for an arbitrary set of states $\mathbb{A}$, the value $\tau^{\mathcal{C},\mathbb{A},t}(s)$ is the probability that $\mathbb{A}$ is reached from s at the time t. Without going into details the vector $\tau^{\mathcal{C},\mathbb{A},t}$ can be computed in a very similar way using the uniformised DTMC $\mathsf{unif}(\mathcal{C})$ as in the case of vector $\pi^{\mathcal{C},s,t}$. Only vector-matrix multiplications are replaced by matrix-transposed-vector multiplication and $\tau^{\mathcal{C},\mathbb{A},0}(s) = 1$ if $s \in \mathbb{A}$, and 0, otherwise.

The global model checking technique cannot be used if Φ includes the operator $\mathsf{E}_{\sim r}[I^{=t}]$. In such a case we have to compute the value $Post(\pi^{\mathcal{C},s,t})$. Hence the local model checking technique has to be employed, i.e., we first compute the vector $\pi^{\mathcal{C},s,t}$ and then apply the user specified function *Post*.

Now we briefly show how the vector $\overline{Prob}^{\mathcal{C},\phi}$ is computed using backward transient analysis. Since the definition of next operator $\mathsf{X}\ \Phi$ does not rely on any real time aspects of CTMCs, its evaluation stems from the probability of the next reaction that can be easily obtained from the transition matrix $\mathbf{R}$. The evaluation of the until operator $\Phi_1 \mathsf{U}^I \Phi_2$ depends on the form of the interval I and is separately solved for the cases of $I = [0, t_1]$ and $I = [t_1, t_2]$ where $t_1, t_2 \in \mathbb{R}_{\geq 0}$. It is based on a modification of the uniformised infinitesimal generator matrix $\mathbf{Q}^{\mathsf{unif}}$ where certain states are made absorbing. This means that all outgoing transitions are ignored reflecting the validity of Φ_1 and Φ_2 in these states.

For any CSL formula Φ, let $\mathcal{C}[\Phi] = (\mathbb{S}, s_0, \mathbf{R}[\Phi], L)$, where $\mathbf{R}[\Phi](s, s') = \mathbf{R}(s, s')$, if $s \vDash \Phi$, and 0, otherwise. The formula $\phi = \Phi_1\ \mathsf{U}^{[0,t]}\ \Phi_2$ can be evaluated using the vector $\tau^{\mathcal{C},\mathbb{A},t}$ in the following way:

$$\overline{Prob}^{\mathcal{C},\phi} = \tau^{\mathcal{C}[\neg\Phi_1 \wedge \Phi_2],\mathbb{A},t} \text{ where } s \in \mathbb{A} \text{ iff } s \vDash \Phi_2.$$

For the formula $\phi = \Phi_1\ \mathsf{U}^{[t_1,t_2]}\ \Phi_2$ the evaluation is split into two parts: staying in states satisfying Φ_1 until time t_1 and reaching a state satisfying Φ_2, while remaining in states satisfying Φ_1, within time $t_2 - t_1$. The formula ϕ can be evaluated using the vector $\tau^{\mathcal{C},\overline{v},t}$ that takes a vector $\overline{v}$ instead of a set $\mathbb{A}$ (i.e., $\tau^{\mathcal{C},\overline{v},0} = \overline{v}$) in the following way:

$$\overline{Prob}^{\mathcal{C},\phi} = \tau^{\mathcal{C}[\neg\Phi_1],\overline{v},t_1} \text{ where } \overline{v} = \tau^{\mathcal{C}[\neg\Phi_1 \wedge \Phi_2],\mathbb{A},t_2-t_1} \text{ and } s \in \mathbb{A} \text{ iff } s \vDash \Phi_2.$$

The backward transient analysis can be also used in the case of reward computation. Since operator $\mathsf{R}_{\sim p}[\mathsf{I}^{=t}]$ expresses the expected reward at time t, the vector $\overline{Exp}^{\mathcal{C},\mathsf{X}_{\mathsf{I}=t}}$ can be computed as follows:

$$\overline{Exp}^{\mathcal{C},\mathsf{X}_{\mathsf{I}=t}} = \tau^{\mathcal{C},\overline{v},t} \text{ where } \overline{v} = \rho \text{ such that } \rho \text{ is the given state reward structure.}$$

For evaluation of the operator $\mathsf{R}_{\sim p}[\mathsf{C}^{\leq t}]$ we have to use *mixed Poisson probabilities* (see, e.g., [2, 10]) in the backward transient analysis. It means that during the uniformisation the Poisson probabilities $\gamma_{i,q \cdot t}$ are replaced by the mixed Poisson probabilities $\bar{\gamma}_{i,q \cdot t}$ that can be computed as:

$$\bar{\gamma}_{i,q \cdot t} = \frac{1}{q} \cdot \left(1 - \sum_{j=1}^{i} \gamma_{j,q \cdot t}\right).$$

Using the given state reward structure ρ we can compute the vector $\overline{Exp}^{\mathcal{C},\mathsf{X}_{\mathsf{C}\leq t}}$ in the following way:

$$\overline{Exp}^{\mathcal{C},\mathsf{X}_{\mathsf{C}\leq t}} = \tau^{\mathcal{C},\overline{v},t}, \text{ where } \overline{v} = \rho \text{ and the mixed Poisson probabilities } \bar{\gamma}_{i,q \cdot t} \text{ are used.}$$

To recap the overall method of stochastic model checking of CTMCs over CSL formulae we present the methods from an abstract perspective. The evaluation of a structured formula Φ proceeds by bottom-up evaluation of a set of atomic propositions, probabilistic or expected reward inequalities and their boolean combinations. This evaluation gives us a discrete set of states that are further used in the following computation. The process continues up the formula until the root is reached. The final verdict is reported either in the form of a boolean yes/no answer or as the actual numerical value of the probability or the expected reward. This process can be easily extended for the operator $\mathsf{E}_{\sim r}[I^{=t}]$, though the local model checking method has to be used.

IV Min-Max Approximation

The key idea of min-max approximation proposed in [11] is to approximate the *largest set of states satisfying* Φ, and the *smallest set of states satisfying* Φ with respect to the space of perturbations $\mathbf{P}$.

Let $\mathbf{C}$ be a set of parameterised CTMCs induced by the space of perturbations $\mathbf{P}$. We compute the approximation $Sat^{\top}_{\mathbf{C}}(\Phi)$ and $Sat^{\perp}_{\mathbf{C}}(\Phi)$ such that

$$Sat^{\top}_{\mathbf{C}}(\Phi) \supseteq \bigcup_{\mathcal{C}_p \in \mathbf{C}} Sat_{\mathcal{C}_p}(\Phi) \;\wedge\; Sat^{\perp}_{\mathbf{C}}(\Phi) \subseteq \bigcap_{\mathcal{C}_p \in \mathbf{C}} Sat_{\mathcal{C}_p}(\Phi),$$

where $s \in Sat_{\mathcal{C}_p}(\Phi)$ iff $s \vDash \Phi$ in CTMC $\mathcal{C}_p$. To obtain such approximations we extended the satisfaction relation $\vDash$ and showed in that it is sufficient for an arbitrary path formula ϕ, and $\mathsf{X} \in \{\mathsf{X}_{\mathsf{C}^{\leq t}}, \mathsf{X}_{\mathsf{I}^{=t}}\}$ to compute the vectors $\overline{Prob}^{\mathbf{C},\phi}_{\top}$, $\overline{Prob}^{\mathbf{C},\phi}_{\perp}$, $\overline{Exp}^{\mathbf{C},\mathsf{X}}_{\top}$ and $\overline{Exp}^{\mathbf{C},\mathsf{X}}_{\perp}$ such that for each $s \in \mathbb{S}$ the following holds:

$$\begin{aligned} \overline{Prob}^{\mathbf{C},\phi}_{\top}(s) &\geq max\{\overline{Prob}^{\mathcal{C}_p,\phi}(s) \mid \mathcal{C}_p \in \mathbf{C}\} \\ \overline{Prob}^{\mathbf{C},\phi}_{\perp}(s) &\leq min\{\overline{Prob}^{\mathcal{C}_p,\phi}(s) \mid \mathcal{C}_p \in \mathbf{C}\} \\ \overline{Exp}^{\mathbf{C},\mathsf{X}}_{\top}(s) &\geq max\{\overline{Exp}^{\mathcal{C}_p,\mathsf{X}}(s) \mid \mathcal{C}_p \in \mathbf{C}\} \text{ for } \mathsf{X} \in \{\mathsf{X}_{\mathsf{I}^{=t}}, \mathsf{X}_{\mathsf{C}^{\leq t}}\} \\ \overline{Exp}^{\mathbf{C},\mathsf{X}}_{\perp}(s) &\leq min\{\overline{Exp}^{\mathcal{C}_p,\mathsf{X}}(s) \mid \mathcal{C}_p \in \mathbf{C}\} \text{ for } \mathsf{X} \in \{\mathsf{X}_{\mathsf{I}^{=t}}, \mathsf{X}_{\mathsf{C}^{\leq t}}\}. \end{aligned} \tag{2}$$

The min-max approximation can be easily extended to the operator $\mathsf{E}_{\sim r}[I^{=t}]$. For the given state $s \in \mathbb{S}$ and the time t it is sufficient to compute the values $Post^{\mathbf{C}}_{\top}(s,t)$ and $Post^{\mathbf{C}}_{\top}(s,t)$ such that the following holds:

$$\begin{aligned} Post^{\mathbf{C}}_{\top}(s,t) &\geq max\{Post(\pi^{\mathcal{C}_p,s,t}) \mid \mathcal{C}_p \in \mathbf{C}\} \\ Post^{\mathbf{C}}_{\perp}(s,t) &\leq min\{Post(\pi^{\mathcal{C}_p,s,t}) \mid \mathcal{C}_p \in \mathbf{C}\}. \end{aligned} \tag{3}$$

The approximated sets $Sat^{\top}_{\mathbf{C}}(\Phi)$ and $Sat^{\perp}_{\mathbf{C}}(\Phi)$ are further used in the computation of $D^{\mathcal{C}}_{\Phi,\mathbf{P},\top}$ and $D^{\mathcal{C}}_{\Phi,\mathbf{P},\perp}$. If the topmost operator of the formula Φ is $\mathsf{P}_{=?}[\phi]$ then

$$D^{\mathcal{C}}_{\Phi,\mathbf{P},\perp} = \overline{Prob}^{\mathbf{C},\phi}_{\perp}(s) \wedge D^{\mathcal{C}}_{\Phi,\mathbf{P},\top} = \overline{Prob}^{\mathbf{C},\phi}_{\top}(s).$$

If the topmost operator of the formula Φ is $\mathsf{R}_{=?}[\mathsf{C}^{\leq t}]$ and $\mathsf{R}_{=?}[\mathsf{I}^{=t}]$ then

$$D^{\mathcal{C}}_{\Phi,\mathbf{P},\perp} = \overline{Exp}^{\mathbf{C},\mathsf{X}}_{\perp}(s) \wedge D^{\mathcal{C}}_{\Phi,\mathbf{P},\top} = \overline{Exp}^{\mathbf{C},\mathsf{X}}_{\top}(s) \text{ for } \mathsf{X} = \mathsf{X}_{\mathsf{C}^{\leq t}} \text{ and } \mathsf{X} = \mathsf{X}_{\mathsf{I}^{=t}}, \text{ respectively.}$$

Similarly, if the topmost operator of the formula Φ is $\mathsf{E}_{=?}[\mathsf{I}^{=t}]$ then

$$D^{\mathcal{C}}_{\Phi,\mathbf{P},\perp} = Post^{\mathbf{C}}_{\perp}(s,t) \wedge D^{\mathcal{C}}_{\Phi,\mathbf{P},\top} = Post^{\mathbf{C}}_{\top}(s,t).$$

V Parameterised Uniformisation

The parameterised uniformisation introduced in [11] is a modification of the standard uniformisation technique that allows us to compute strict approximations of the minimal and maximal transient probability with respect to the set $\mathbf{C}$, moreover, the modification preserves the asymptotic time complexity of standard uniformisation. For the given state $s \in \mathbb{S}$ and time $t \in \mathbb{R}_{\geq 0}$ the parameterised uniformisation returns vectors $\pi^{\mathbf{C},s,t}_{\top}$ and $\pi^{\mathbf{C},s,t}_{\perp}$ such that for each state $s' \in \mathbb{S}$ the following holds:

$$\pi^{\mathbf{C},s,t}_{\top}(s') \geq max\{\pi^{\mathcal{C}_p,s,t}(s') \mid \mathcal{C}_p \in \mathbf{C}\} \;\wedge\; \pi^{\mathbf{C},s,t}_{\perp}(s') \leq min\{\pi^{\mathcal{C}_p,s,t}(s') \mid \mathcal{C}_p \in \mathbf{C}\}$$

The modification is based on the computation of the local maximum (minimum) of $\pi^{\mathcal{C}_p,s,t}(s')$ over all $\mathcal{C}_p \in \mathbf{C}$ for each state *s'* and in each iteration i of the standard uniformisation. It means that in the ith iteration of the computation for a state *s'* we consider only the maximal (minimal) values in the relevant states in the iteration *i-1*, i.e., the states that affect $\pi^{\mathcal{C}_p,s,t}(s')$.

In [11] we have defined the function $\sigma(s)$ (formally $\sigma(p,s,\pi)$) which for each state $s \in \mathbb{S}$, perturbation point $p \in \mathbf{P}$, and probability distribution π (or pseudo-distribution with the sum smaller or larger than

1) returns the difference of probability mass inflow and outflow to/from state s. If all reactions are described by mass action kinetics the resulting σ functions are monotonic with respect to any single perturbed stochastic rate constant k_r. This allows us to efficiently compute for each state s' the local maximum (minimum) of $\pi^{\mathcal{C}_p,s,t}(s')$ over all $\mathcal{C}_p \in \mathbf{C}$ corresponding to $\mathbf{P}$.

However, in the case of more complex rate functions than those resulting from mass action kinetics, the corresponding $\sigma(s)$ function does not have to be in general monotonic over $k_r \in [k_r^{\perp}, k_r^{\top}]$ for all states s. This makes the computation of local extremes with respect to k_r more complex, though still tractable. In the following let us assume the space of perturbations $\mathbf{P} = [k_r^{\perp}, k_r^{\top}] \times \mathbf{P}'$ is be decomposed along the k_r axis.

The key idea is for each state s to be able to efficiently decompose $\mathbf{P}$ into subspaces $\mathbf{P} = \mathbf{P}_1 \cup \ldots \cup \mathbf{P}_n$, such that for each $\mathbf{P}_i$ the function $\sigma(s)$ over $\mathbf{P}_i$ is monotonic and then use the original method. The problem is that a computation of such a strict decomposition into monotonic subspaces is computationally demanding. Therefore we use a simplification, by off-line functional analysis we identify properties of σ functions for a given class of reaction kinetics and then obtain a partial decomposition of $\mathbf{P}$ based on function derivations into subspaces where monotonicity is guaranteed. For the remaining subspaces $\mathbf{P}_j$, where monotonicity of σ is not guaranteed, we employ a less accurate approximation.

We decompose the function $\sigma(s)$ over $\mathbf{P}_j$ into functions $\alpha_k^{s,\mathbf{P}_j}$ and $\beta_l^{s,\mathbf{P}_j}$ such that:

$$\sigma(s) = \sum_{k=1}^{K} \alpha_k^{s,\mathbf{P}_j} - \sum_{l=1}^{L} \beta_l^{s,\mathbf{P}_j}$$

and each $\alpha_k^{s,\mathbf{P}_j}$ and $\beta_l^{s,\mathbf{P}_j}$ is monotonic. This allows us to use the original method to compute the maximum and minimum of the functions $\alpha_k^{s,\mathbf{P}_j}$ and $\beta_l^{s,\mathcal{P}_j}$ over the interval $\mathbf{P}_j$, denoted as $max(\alpha_k^{s,\mathbf{P}_j})$, $min(\alpha_k^{s,\mathbf{P}_j})$, $max(\beta_k^{s,\mathbf{P}_j})$ and $min(\beta_k^{s,\mathbf{P}_j})$, respectively. Note that this decomposition can be easily obtained from the definition of the rate function f_r. Now the maximum and minimum of $\sigma(p,s)$ over $\mathbf{P_j}$ can be approximated in the following way:

$$max\{\sigma(p,s) \mid p \in \mathbf{P}_j\} \leq \sum_{k=1}^{K} max(\alpha_k^{s,\mathbf{P}_j}) - \sum_{l=1}^{L} min(\beta_l^{s,\mathbf{P}_j})$$

$$min\{\sigma(p,s) \mid p \in \mathbf{P}_i\} \geq \sum_{k=1}^{K} min(\alpha_k^{s,\mathbf{P}_i}) - \sum_{l=1}^{L} max(\beta_l^{s,\mathbf{P}_j}).$$

This approximation increases the inaccuracy of parameterised uniformisation, however, the subspaces $\mathbf{P}_j$ where the monotonicity of $\sigma(s)$ is not guaranteed are usually small and, together with perturbation space decomposition introduced in the following section, they keep on getting smaller. Hence, the additional inaccuracy of the presented extension is manageable. Despite the fact that the time demands of this approximation are orders of magnitudes lower than other numerical methods computing maximum/minimum of $\sigma(s)$ over $\mathbf{P}_i$, they still significantly slow down the computation of parameterised uniformisation.

The aforementioned parameterised uniformisation can be straightforwardly employed also for backward transient analysis. It means that we can efficiently compute the vectors $\tau_{\top}^{\mathbf{C},\mathbb{A},t}$ and $\tau_{\perp}^{\mathbf{C},\mathbb{A},t}$ such that for the given set of states $\mathbb{A}$ and each state $s \in \mathbb{S}$ the following holds:

$$\tau_{\top}^{\mathbf{C},\mathbb{A},t}(s) \geq max\{\tau^{\mathcal{C}_p,\mathbb{A},t}(s) \mid \mathcal{C}_p \in \mathbf{C}\} \ \wedge \ \tau_{\perp}^{\mathbf{C},\mathbb{A},t}(s) \leq min\{\tau^{\mathcal{C}_p,\mathbb{A},t}(s) \mid \mathcal{C}_p \in \mathbf{C}\}$$

Once we know how to compute the vectors $\tau_{\top}^{\mathbf{C},\mathbb{A},t}$ and $\tau_{\perp}^{\mathbf{C},\mathbb{A},t}$ the global model checking technique for non-parameterised CTMCs can be directly employed. To obtain the vectors $\overline{Prob}_{\top}^{\mathbf{C},\phi}$, $\overline{Prob}_{\perp}^{\mathbf{C},\phi}$, $\overline{Exp}_{\top}^{\mathbf{C},\mathsf{X}}$ and $\overline{Exp}_{\perp}^{\mathbf{C},\mathsf{X}}$ satisfying Equation 2, it is sufficient to replace the backward transient distribution $\tau_{\perp}^{\mathcal{C},\mathbb{A},t}$ by

the vectors $\tau_{\top}^{\mathbf{C},\mathbb{A},t}$ and $\tau_{\top}^{\mathbf{C},\mathbb{A},t}$. However for a general class of user-defined post-processing functions *Post*, the vectors $\pi_{\top}^{\mathbf{C},s,t}$ and $\pi_{\bot}^{\mathbf{C},s,t}$ cannot be directly used to compute values of $Post_{\top}^{\mathbf{C}}(s,t) = Post(\pi_{\top}^{\mathbf{C},s,t})$ nor $Post_{\bot}^{\mathbf{C}}(s,t) = Post(\pi_{\bot}^{\mathbf{C},s,t})$ that would satisfy Equation 3, since there is no guarantee about the projective properties of the function *Post*.

Now we show the main idea of computing $Post_{\top}^{\mathbf{C}}(s,t)$ and $Post_{\bot}^{\mathbf{C}}(s,t)$ for the post-processing function *Post* defined as the mean quadratic deviation of a probability distribution. This function allows us to quantify and analyse noise in different variants of signalling pathways that are studied in the second case study. The post-processing function is defined as $Post(\pi) = \sum_{s\in\mathbb{S}} |s(A) - mean(\pi, A)|^2 \cdot \pi(s)$, where $s(A)$ gives the population of A in state s and $mean(\pi, A)$ is the mean of the distribution π defined as $mean(\pi, A) = \sum_{s\in\mathbb{S}} s(A) \cdot \pi(s)$.

Let us suppose we have an upper and lower bound on the probability distribution $\pi_{\top}, \pi_{\bot}$ obtained by the parameterised uniformisation. It means that $\forall \mathcal{C}_p \in \mathbf{C}$ and $\forall s \in \mathbb{S}.\ \pi_{\bot}(s) \leq \pi^{\mathcal{C}_p}(s) \leq \pi_{\top}(s)$. To find the maximal value $max\left\{Post(\pi^{\mathcal{C}_p}) \mid \mathcal{C}_p \in \mathbb{C}\right\}$ means to find the distribution π^{max} such that $\sum_{s\in\mathbb{S}} \pi^{max}(s) = 1$, $\forall s \in \mathbb{S}.\ \pi_{\bot}(s) \leq \pi^{max}(s) \leq \pi_{\top}(s)$ and the probability mass in π^{max} is distributed with the farthest distance from the mean. Clearly, such a distribution has a maximal mean quadratic deviation. Note that the number of distributions satisfying the first two conditions is uncountable. Thus we cannot employ a direct search strategy.

Our searching strategy builds on the observation that only the distributions that localise most of the mass as far as possible from the mean (i.e., maximising the mean quadratic deviation and still meeting the bounds $\pi_{\bot}, \pi_{\top}$), have to be considered. These distributions can be linearly ordered with respect to the sum of mass x localised at the low population end of the state space. It can be shown that the function that evaluates *Post* on all these distributions is piece-wise quadratic with respect to x and has $O(|\mathbb{S}|)$ segments. Therefore, $O(|\mathbb{S}|)$ many steps are sufficient to compute $max\left\{Post(\pi^{\mathcal{C}_p}) \mid \mathcal{C}_p \in \mathbb{C}\right\}$.

To compute the minimal value $min\left\{Post(\pi^{\mathcal{C}_p}) \mid \mathcal{C}_p \in \mathbb{C}\right\}$ we proceed analogously, i.e., only the distributions that localise most of the mass as close as possible to the mean are considered. This leads again to a piece-wise quadratic function. It is also important to note that the perturbation space decomposition presented in the next section allows us to obtain the values $Post_{\top}^{\mathbf{C}}(s,t)$ and $Post_{\bot}^{\mathbf{C}}(s,t)$ with the desired precision.

VI Perturbation Space Decomposition Strategy

For the sake of simplicity, we present the parametric decomposition strategy only on the computation of $\pi_{\star}^{\mathbf{C},s,t}$ and $\tau_{\star}^{\mathbf{C},\mathbb{A},t}$ for $\star \in \{\top, \bot\}$ and $\mathbf{P}$, since it can be easily extended to the computation of $D_{\Phi,\mathbf{P},\star}^{\mathcal{C}}$ for any formula Φ. The key part of the parametric decomposition is to decide when the inspected subspace should be further decomposed. The condition for the decomposition is different for $\pi_{\star}^{\mathbf{C},s,t}$ and $\tau_{\star}^{\mathbf{C},\mathbb{A},t}$. Since the vector $\pi_{\star}^{\mathbf{C},s,t}$ gives us the transient probability distribution from the state s that is further used to compute $D_{\Phi,\mathbf{P},\star}^{\mathcal{C}}$, we consider the following condition. The space $\mathbf{P}$ is decomposed if during the computation of parameterised uniformisation in an iteration i it holds that:

$$\sum_{k=1}^{|\mathbb{S}|} \pi_{\top}^{\mathbf{C},s,i}(s_k) - \sum_{k=1}^{|\mathbb{S}|} \pi_{\bot}^{\mathbf{C},s,i}(s_k) > \textsc{Err}$$

where $\pi_{\star}^{\mathbf{C},s,i}$ denotes the corresponding approximation of $\pi^{\mathbf{C},s,0} \cdot (\mathbf{Q}^{\mathsf{unif}(\mathbf{C})})^i$.

In contrast to $\pi_{\star}^{\mathbf{C},s,t}$, the value $\tau_{\top}^{\mathbf{C},\mathbb{A},t}(s)$ for each state $s \in \mathbb{S}$ is further used to $D_{\Phi,\mathbf{P},\star}^{\mathcal{C}}$ and thus we consider a different condition. The space $\mathbf{P}$ is decomposed if during the computation of parameterised uniformisation in an iteration i for any state s it holds that:

$$\tau_{\top}^{\mathbf{C},\mathbb{A},i}(s) - \tau_{\bot}^{\mathbf{C},\mathbb{A},i}(s) > \textsc{Err}.$$

If the decomposition takes place we cancel the current computation and decompose the perturbation space $\mathbf{P}$ to n subspaces such that $\mathbf{P} = \mathbf{P}_1 \cup \ldots \cup \mathbf{P}_n$. Each subspace $\mathbf{P}_j$ defines a new set of CTMCs $\mathbf{C}_j = \{\mathcal{C}_j \mid j \in \mathbf{P}_j\}$ that is independently processed in a new computation branch. Note that we could reuse the previous computation and continue from the iteration $i-1$. However, the most significant part of the error is usually cummulated during the previous iterations and thus the decomposition would have only a negligible impact on error reduction.

A minimal decomposition with respect to the perturbation space $\mathbf{P}$ defines a minimal number of subspaces m such that $\mathbf{P} = \mathbf{P}_1 \cup \ldots \cup \mathbf{P}_m$ and for each subspace $\mathbf{P}_j$, where $1 \leq j \leq m$, it holds that $Err^{\mathcal{C},s}_{\Phi,\mathbf{P}_j} \leq \textsc{Err}$, where $Err^{\mathcal{C},s}_{\Phi,\mathbf{P}_j} = D^{\mathcal{C}}_{\Phi,\mathbf{P}_j,\top} - D^{\mathcal{C}}_{\Phi,\mathbf{P}_j,\bot}$. Note that the existence of such decomposition is guaranteed only if the evaluation function $D^{\mathcal{C},s}_{\Phi}$ over $\mathbf{P}$ is continuous. If the evaluation function is continuous there can exist more than one minimal decomposition. However, it cannot be straightforwardly found. To overcome this problem we have considered and implemented several heuristics allowing to iteratively compute a decomposition satisfying the following: (1) it ensures the required error bound whenever $D^{\mathcal{C}}_{\Phi}$ over $\mathbf{P}$ is continuous, (2) it guarantees the refinement termination in the situation where $D^{\mathcal{C},s}_{\Phi}$ over $\mathbf{P}$ is not continuous and the discontinuity causes that Err cannot be achieved. To ensure the termination an additional parameter has to be introduced as a lower bound on the subspace size. Hence this parameter provides a supplementary termination criterion.

References

[1] Kwiatkowska M, Norman G, Parker D (2011) PRISM 4.0: Verification of probabilistic real-time systems. In: Computer Aided Verification. Springer, volume 6806 of *LNCS*, pp. 585–591.

[2] Kwiatkowska M, Norman G, Parker D (2007) Stochastic model checking. In: Formal Methods for Performance Evaluation, Springer, volume 4486 of *LNCS*. pp. 220–270. doi:10.1007/978-3-540-72522-0_6.

[3] Fox BL, Glynn PW (1988) Computing Poisson probabilities. Commun ACM 31: 440–445.

[4] Munsky B, Khammash M (2006) The finite state projection algorithm for the solution of the chemical master equation. The Journal of chemical physics 124: 044104.

[5] Henzinger TA, Mateescu M, Wolf V (2009) Sliding Window Abstraction for Infinite Markov Chains. In: Computer Aided Verification, Springer, volume 5643 of *LNCS*. pp. 337-352. doi:10.1007/978-3-642-02658-4_27.

[6] Didier F, Henzinger TA, Mateescu M, Wolf V (2009) Fast Adaptive Uniformization of the Chemical Master Equation. In: High Performance Computational Systems Biology. IEEE Computer Society, pp. 118–127.

[7] Zhang J, Watson LT, Cao Y (2009) Adaptive aggregation method for the chemical master equation. International Journal of Computational Biology and Drug Design 2: 134-148.

[8] van Moorsel APA, Sanders WH (1994) Adaptive uniformization. ORSA Communications in Statistics: Stochastic Models, vol 10, no 3 : 619–648.

[9] Baier C, Haverkort B, Hermanns H, Katoen JP (2000) Model Checking Continuous-Time Markov Chains by Transient Analysis. In: Computer Aided Verification, Springer, volume 1855 of *LNCS*. pp. 358-372. doi:10.1007/10722167_28.

[10] Kwiatkowska M, Norman G, Pacheco A (2006) Model Checking Expected Time and Expected Reward Formulae with Random Time Bounds. Computers & Mathematics with Applications 51: 305 - 316.

[11] Brim L, Češka M, Dražan S, Šafránek D (2013) Exploring parameter space of stochastic biochemical systems using quantitative model checking. In: Computer Aided Verification. Springer Berlin Heidelberg, volume 8044 of *LNCS*, pp. 107-123. doi:10.1007/978-3-642-39799-8_7.
